# A catalog of validity indices for step counting wearable technologies during treadmill walking: the CADENCE-Kids study

**DOI:** 10.1186/s12966-021-01167-y

**Published:** 2021-07-16

**Authors:** Zachary R. Gould, Jose Mora-Gonzalez, Elroy J. Aguiar, John M. Schuna, Tiago V. Barreira, Christopher C. Moore, John Staudenmayer, Catrine Tudor-Locke

**Affiliations:** 1 Department of Kinesiology, University of Massachusetts Amherst, Amherst, MA USA; 2 College of Health and Human Services, University of North Carolina at Charlotte, 9201 University City Blvd., Charlotte, NC 28223 USA; 3 Department of Kinesiology, The University of Alabama, Tuscaloosa, AL USA; 4 School of Biological and Population Health Sciences, Oregon State University, Corvallis, OR USA; 5 Exercise Science Department, Syracuse University, Syracuse, NY USA; 6 Department of Epidemiology, University of North Carolina at Chapel Hill, Chapel Hill, NC USA; 7 Department of Mathematics and Statistics, University of Massachusetts Amherst, Amherst, MA USA

**Keywords:** Accelerometer, Accuracy, Bias, Measurement, Pedometer, Physical activity

## Abstract

**Background:**

Wearable technologies play an important role in measuring physical activity (PA) and promoting health. Standardized validation indices (i.e., accuracy, bias, and precision) compare performance of step counting wearable technologies in young people.

**Purpose:**

To produce a catalog of validity indices for step counting wearable technologies assessed during different treadmill speeds (slow [0.8–3.2 km/h], normal [4.0–6.4 km/h], fast [7.2–8.0 km/h]), wear locations (waist, wrist/arm, thigh, and ankle), and age groups (children, 6–12 years; adolescents, 13–17 years; young adults, 18–20 years).

**Methods:**

One hundred seventeen individuals (13.1 ± 4.2 years, 50.4% female) participated in this cross-sectional study and completed 5-min treadmill bouts (0.8 km/h to 8.0 km/h) while wearing eight devices (Waist: Actical, ActiGraph GT3X+, NL-1000, SW-200; Wrist: ActiGraph GT3X+; Arm: SenseWear; Thigh: activPAL; Ankle: StepWatch). Directly observed steps served as the criterion measure. Accuracy (mean absolute percentage error, MAPE), bias (mean percentage error, MPE), and precision (correlation coefficient, *r*; standard deviation, SD; coefficient of variation, CoV) were computed.

**Results:**

Five of the eight tested wearable technologies (i.e., Actical, waist-worn ActiGraph GT3X+, activPAL, StepWatch, and SW-200) performed at < 5% MAPE over the range of normal speeds. More generally, waist (MAPE = 4%), thigh (4%) and ankle (5%) locations displayed higher accuracy than the wrist location (23%) at normal speeds. On average, all wearable technologies displayed the lowest accuracy across slow speeds (MAPE = 50.1 ± 35.5%), and the highest accuracy across normal speeds (MAPE = 15.9 ± 21.7%). Speed and wear location had a significant effect on accuracy and bias (*P* < 0.001), but not on precision (*P* > 0.05). Age did not have any effect (*P >* 0.05).

**Conclusions:**

Standardized validation indices focused on accuracy, bias, and precision were cataloged by speed, wear location, and age group to serve as important reference points when selecting and/or evaluating device performance in young people moving forward. Reduced performance can be expected at very slow walking speeds (0.8 to 3.2 km/h) for all devices. Ankle-worn and thigh-worn devices demonstrated the highest accuracy. Speed and wear location had a significant effect on accuracy and bias, but not precision.

**Trial registration:**

Clinicaltrials.govNCT01989104. Registered November 14, 2013.

**Supplementary Information:**

The online version contains supplementary material available at 10.1186/s12966-021-01167-y.

## Introduction

Physical activity (PA) is a powerful marker of health throughout the lifespan, including during the developmental transition from childhood to adolescence and on to young adulthood [1–3]. The surge in contemporary wearable technologies that produce step counting outputs offers an important opportunity to promote the health-related benefits associated with PA in young people [4–6]. To this end, the 2018 U.S. Physical Activity Guidelines Advisory Committee Scientific Report [7] advocated for conducting more research in order to translate public health PA guidelines using step-based metrics (e.g., steps/day, steps/min) that can be widely used and easily interpreted by researchers, clinicians, and consumers [8]. Available wearable technologies vary greatly in terms of their step-counting mechanism/algorithms, resulting in differential sensitivity and specificity to step measurement [9, 10]. In addition, there are important external factors, or threats to validity, that may also influence device (wearable) performance, such as speed or wear location [11]. An additional potential threat to validity is age, since children do not necessarily walk in a manner that is identical to adults’ gait patterns [12–14]. For example, 7-year-old children lack neuromuscular maturity, especially at the ankle (i.e., diminished peak plantar flexor moment and reduced peak power absorption and generation), to produce an adult-like gait [15]. Also, children (5–9 years of age) walked with significantly different step time, cycle time, cycle frequency and cadence than their adult peers (19–32 years of age) in walking and running tests [16]. It is therefore critical to consider such threats to validity when comparing performance of step counting wearable technologies using standardized methods and validation metrics among a cohort of children [17].

The Consumer Technology Association (CTA) released guidance in 2016 for treadmill-based validation assessment of step counting wearable technologies [18]. The CTA suggested that, under controlled laboratory conditions, direct observation and video backup should be the criterion measures. The performance of step counting wearable technologies should then be compared to this criterion standard [18]. Standardized and harmonized validation indices, specifically accuracy, bias, and precision are also necessary to facilitate comparability of different types of wearable technologies [17, 19, 20]. The CTA [18], Welk et al. [20], Walther et al. [19], and a previous scoping review from our research group [17], recommended that accuracy (defined as the overall distance between estimated or observed values and the true value [19]) be determined using mean absolute percentage error (MAPE,), calculated as follows:

$$ {E}_j={W}_j-{C}_j $$

$$ \mathrm{MAPE}=\frac{100\%}{n}{\sum}_{j=1}^n\frac{\left|{E}_j\right|}{C_j} $$

Where *W*_*j*_ is the number of steps recorded by the wearable technology being tested in the *j*^th^ person-bout (*j* = 1, 2, …, *n*), *C*_*j*_ is the criterion standard of observed steps in that same person-bout, and *E*_*j*_ is the corresponding step count error that is expressed in absolute terms. We are aware of the different uses of this terminology before. For example, like root mean squared error (RMSE), it can be demonstrated that MAPE is also a measure of both bias (the inverse of accuracy) and variance (the inverse of precision). Herein we follow the work of Welk et al. [20] who advocated for measuring accuracy using MAPE and the CTA guidelines [18] which also used MAPE in this capacity. Bias refers to magnitude and direction (i.e., over or underestimations) of systematic errors [19]. It can be visually presented in Bland-Altman plots, but this graphical format makes it difficult to compare across studies. Alternatively, bias can be represented and compared numerically using mean percentage error (MPE). MPE is calculated as follows [21]:

$$ \mathrm{MPE}=\frac{100\%}{n}{\sum}_{j=1}^n\frac{E_j}{C_j} $$

By dividing the difference of the steps derived from wearable technology and the directly observed steps (*E*_*j*_) by the observed steps (*C*_*j*_), the result is a scaled index that explains the difference, regardless of the total number of steps taken. Also, it can discriminate between estimation methods that, on average, over-estimate or under-estimate the criterion. Precision is defined as the assessment of random error (i.e., that due to chance, or just naturally occurring), and is commonly referred to as variance or variability [19]. It represents variability in the difference between directly observed steps and steps derived from wearable technology, and is generally reported as standard deviation (SD) of error values (*E*) [19]:

$$ \mathrm{SD}=\sqrt{\ \frac{1}{\mathrm{n}}{\sum}_{\mathrm{j}=1}^{\mathrm{n}}{\left({\mathrm{E}}_{\mathrm{j}}-\overline{E}\right)}^2} $$

Another precision index is coefficient of variation (CoV), calculated as:

$$ \mathrm{CoV}=\left(\frac{\mathrm{SD}}{\overline{E}}\right)\times 100\% $$

Where *SD* is the variance of the steps derived from wearable technology, and $\overline{E}$ represents the average of errors. Also, precision can be reported as a correlation coefficient (*r*) that indicates the strength of the relationship between directly observed steps and steps derived from wearable technology:

$$ r=\frac{\sum_{j=1}^n\left({W}_j-\overline{W}\right)\left({C}_j-\overline{C}\right)}{\sqrt{\left[{\sum}_{j=1}^n{\left({W}_j-\overline{W}\right)}^2\right]\left[{\sum}_{j=1}^n{\left({C}_j-\overline{C}\right)}^2\right]}} $$

Where *W*_*j*_ is the number of steps recorded by the wearable technology being tested in the *j*^*th*^ person-bout (j = 1, 2, …, n), and *C*_*j*_ is the criterion standard of observed steps in that same person-bout.

There are only a few youth-specific studies that used direct observation of steps as a criterion standard in treadmill-based studies [21–27] (see Additional file 1 for a summary of these studies). Those that exist used small sample sizes (ranging from 17 to 45 participants) and limited age ranges within the broader childhood to young adulthood age span. Testing protocols varied in the number of treadmill bouts (3 to 5 bouts) and their durations (2-min to 5-min). Tested speeds varied and ranged from 0.8 km/h (0.5 mph) to 9.7 km/h (6.0 mph). Also, none of these studies included all three measures of validity (i.e., accuracy, bias, and precision) or assessed the effect of age on wearable technology step counting performance [17].

The purpose of this secondary analysis of the CADENCE-Kids’ data set [28] was to compute and compare speed, wear location, and age-specific validity indices of accuracy, bias, and precision across eight different wearable technologies evaluated in a broadly-aged (6–20 years of age) sample of children, adolescents and young adults. A valuable product of this effort is a catalog (digital source of reference material and values) intended to better facilitate evaluation and comparison of step counting wearable technologies.

## Methods

### Study design and regulatory information

CADENCE-Kids was a laboratory-based, cross-sectional study designed to establish cadence (steps/min) thresholds associated with absolutely-defined PA intensity across the developmental lifespan of 6–20 years of age [28]. Full descriptions of the primary aim, protocol details, measurements, and inclusion/exclusion criteria can be found in the original study [28]. The protocol was registered at ClinicalTrials.gov (NCT01989104) and conducted at the Pennington Biomedical Research Center in Baton Rouge, Louisiana, United States from January 2014 to April 2015. All original study procedures were reviewed and approved by the Pennington Biomedical Institutional Review Board. Description of the purpose and characteristics of the study was provided to the parents and participants, and child assent and parental permission were obtained from children and adolescents 6–17 years of age. Participants between 18 and 20 years of age provided informed consent. Approval for these secondary analyses was granted by the University of Massachusetts Amherst Institutional Review Board.

### Participants

To minimize important sources of bias, improve the generalizability of findings and ensure a relatively equal distribution of participants across the evaluated age range of this study (6–20 years of age), a balanced sex-and-age distribution of at least 4 boys and 4 girls for each age-year between 6 and 20 years were recruited for a total of 123 children, adolescents and young adults. Briefly, exclusion criteria were: use of wheelchairs or having other impairments for normal ambulation, hospitalized for mental illness within the past 5 years, and medical condition or medication that might affect heart rate or metabolic response to exercise testing or be aggravated by exercise.

### Treadmill testing procedure

Fasted participants (at least 4 h) completed a series of up to ten 5-min walking/running bouts on a level treadmill (0% grade), which started at 0.8 km/h (0.5 mph) and subsequently increased in 0.8 km/h increments up to a maximum of 8.0 km/h (5.0 mph). A complete list of bouts and speed conversions in m/min, km/h, and mph is published elsewhere [28]. Each bout was separated by at least 2-min of standing rest to facilitate collection of bout-specific step counts from the various wearable technologies. The protocol was terminated following the bout when participants naturally chose to jog or run, or if they or the researcher decided not to continue at any time point.

### Measures

#### Participant characteristics and anthropometric measures

Participant’s biological sex, race/ethnicity, and age were self-reported. A series of anthropometric measurements that included weight, standing height, leg length, and body mass index (BMI) calculations were then obtained. Details for these measurements are presented in the original CADENCE-Kids’ reports [28, 29].

#### Step counting

Directly observed steps were obtained using a hand-tally counter for a criterion measure of step counts. A video camera was also directed at the participants’ feet and recorded movements for step verification purposes. Video recordings were referred to in cases that staff-disclosed miscounting or when ambiguous data were identified during data processing.

During the treadmill protocol, participants wore eight step counting wearable technologies across five different wear locations (for tabular and visual presentations of device settings and initialization procedures, see Supplementary Table 1 and Supplementary Figure 1 respectively, Additional file 2). Specifically, they wore an Actical (Philips Respironics, Murrysville, PA, USA) and a New Lifestyles SW-200 (Yamax Corporation, Tokyo, Japan) on the left waist, and an ActiGraph GT3X+ (ActiGraph, Pensacola, FL, USA) and a New Lifestyles NL-1000 (New Lifestyles Inc., Lee’s Summit, MO, USA) on the right hip; an ActiGraph GT3X+ on the non-dominant wrist and a SenseWear Armband (BodyMedia, Inc., Pittsburgh, PA, USA) on the right arm; an activPAL (PAL Technologies Ltd., Glasgow, UK) on the right thigh; and a StepWatch (OrthoCare Innovations, Seattle, WA, USA) on the right ankle.

### Data processing and aggregation

The NL-1000 and SW-200 provided real-time step count data that were recorded at the end of each bout. Notably, only the research staff running the trials examined the displayed step outputs. The other wearable technologies (i.e., Actical, ActiGraph GT3X+, activPAL, SenseWear, and StepWatch) did not display real-time step count feedback, however, data were automatically time-stamped according to internal mechanisms and subsequently downloaded following manufacturers’ protocols (see Supplementary Table 1, Additional file 2). Specifically, the time-stamped step count data were synchronized to the protocol’s digital timing record to inform post-processing of bout-specific step counts. The variables consisted of step counts for each wearable technology at each respective bout. For every participant and for each completed protocol stage, we then merged all step count data from the hand-tally criterion, SW-200, NL-1000, and timestamped wearable devices into a single comma-delimited flat file for further analysis.

### Analytic sample

Of the 123 originally recruited participants, there were 2 who failed screening procedures due to medications, that were revealed after enrollment, which could have adversely affected heart rate and/or metabolism during treadmill testing. Additionally, data were not available for 4 participants due to wearable technologies malfunctioning and data collection errors. Thus, the final analytic data set included 117 participants providing a total of 1008 bouts. Of these, 89 were running bouts distributed across different speeds as follows: 4.8 km/h (*n* = 3), 5.6 km/h (*n* = 6), 6.4 km/h (*n* = 20), 7.2 km/h (*n* = 41), and 8.0 km/h (*n* = 19). Running bouts were subsequently excluded from the present analysis due to the overall lack of a robust sample size in terms of running bouts at each standardized speed and also to the known differences in gait and biomechanical patterns between running and walking [7]. The final analytic dataset and corresponding data dictionary can be accessed in Additional file 3.

### Statistical analysis

#### Descriptive statistics

Sample characteristics are presented as means and SD or percentages (%), as appropriate. A catalog of wearable technologies’ performance (i.e., indices of accuracy, bias, and precision) was calculated and collated. The accuracy (MAPE) and bias (MPE) values with their associated precision indices (SD and CoV) averaged across the sample were determined for each wearable technology and relative to each walking speed bout, each speed level (slow = 0.8, 1.6, 2.4, and 3.2 km/h; normal = 4.0, 4.8, 5.6, and 6.4 km/h; and fast = 7.2 and 8.0 km/h), wear location (waist, wrist/arm, thigh, and ankle), and age group (children, 6–12 years; adolescents, 13–17 years; and young adults, 18–20 years). The classification of speed levels in slow, normal and fast was performed based on the CTA guidance and its definition of the normal speed range [18]. Lower MAPE values are indicative of higher accuracy and MPE values closer to 0% are indicative of improved bias. Correlation coefficients (*r*; another precision index for the strength of the linear relationship between directly observed steps and steps derived from each wearable technology, where *r* closer to 1 is indicative of a stronger, more linear relationship) were computed for the whole sample and reported across all walking bouts. Importantly, this index requires a wide range of step counts to provide meaningful results.

#### Inferential analysis

Inferential analyses were performed to test for the effect of speed, wear location and age on the overall accuracy, bias, and precision. First, we fit a set of eight mixed effects models to examine the effect of speed on MAPE (i.e., accuracy) for each of the eight wearable technologies. Specifically, for each device, the MAPE for participant *i* = 1, 2, …, *N* at speed *j* = 1, 2, …, *q* (included as a categorical variable), conditional on their participant-specific deviation, was estimated using the following model:

$$ E\left[{Y}_i|{b}_i\right]={X}_i\beta +{b}_i $$

Where *Y*_*i*_ was a *q* × 1 vector of absolute percentage error values, *X*_*i*_ was a *q* × *q* diagonal matrix of dummy variables (equal to 0 or 1) indexing the corresponding speed, *β* was a *q* × 1 vector of regression coefficients for the fixed effect (categorical speed), and *b*_*i*_ was the random intercept for participant *i*. Likelihood ratio tests (α = 0.05) were then used to test for the effect of speed (*β*) on MAPE for the wearable technology-specific model. Additionally, we estimated 95% CIs for MAPEs at each speed, and 95% CIs that did not overlap were interpreted as significantly different, while 95% CIs that overlapped with another point estimate were interpreted as not significantly different [30]. This analysis was then repeated to examine the effect of wear location (i.e., waist, wrist/arm, thigh, and ankle) as well age group (i.e., children, adolescents, and young adults) by substituting for *X*_*i*_ and refitting the model separately for each of the three speed levels (i.e., slow, normal, and fast). For example, for the effect of wear location on MAPE at each of the speed levels, *X*_*i*_ was a diagonal matrix of dummy variables (equal to 0 or 1) corresponding to location-speed combinations. Given that accuracy (MAPE) accounts for the overall performance of a step-counting device and thus reflects both bias and precision [19], main analyses of the present study were performed for accuracy (MAPE) of wearable technologies, but analyses were also performed for bias (MPE) and precision (*r*) separately. Thus, all these models were fit again to examine the effects of speed, wear location and age on bias (MPE) and correlation (*r*) (substituting for *Y*_*i*_), and they can all be found in supplementary material (see Results for specific references to these supplementary materials). All descriptive and inferential statistical analyses were conducted using R-Studio (version 3.0.2, R Foundation for Statistical Computing, Vienna, Austria).

## Results

### Descriptive statistics

#### Sample characteristics

Descriptive characteristics of the analytical sample (*N* = 117) are reported in Table 1. The total number of participants who completed each walking bout and the average number of steps derived from direct observation and from any of the wearable technologies at each speed are detailed for the whole sample and by age group in Additional file 4. Of note, no children of 6–12 years of age completed the final possible bout (8.0 km/h).

**Table 1:** Descriptive characteristics of the analytical sample

Variables	All (***N*** = 117)		Children, 6–12 years (***n*** = 53)		Adolescents, 13–17 years (***n*** = 40)		Young Adults, 18–20 years (***n*** = 24)	
	Mean	SD	Mean	SD	Mean	SD	Mean	SD
Age (years)	13.17	4.22	9.21	1.95	14.93	1.40	19.00	0.83
Weight (kg)	55.94	21.85	41.19	14.67	67.97	21.60	68.43	14.60
Height (cm)	155.66	16.47	141.89	12.44	165.29	8.60	170.01	8.92
Leg length (cm)	73.94	9.42	67.12	8.16	79.10	6.46	80.40	5.41
BMI (kg/m^2^)	22.41	6.25	20.04	5.17	24.78	7.15	23.66	5.06
	n	%	n	%	n	%	n	%
Sex (female)	59	50.4	26	49.1	21	52.5	12	50.0
BMI classifications^a^								
Underweight	6	5.1	3	5.7	1	2.5	2	8.3
Normal weight	67	57.3	27	50.9	22	55.0	18	75.0
Overweight	18	15.4	9	17.0	7	17.5	2	8.3
Obese	26	22.2	14	26.4	10	25.0	2	8.3
Race/ethnicity								
African-American	41	35.0	21	39.6	13	32.5	7	29.2
Caucasian	73	62.4	30	56.6	26	65.0	17	70.8
Other	3	2.6	2	3.8	1	2.5	0	0.0

*BMI* body mass index. ^a^BMI classifications defined as BMI < 5th percentile (underweight), 5th ≤ BMI < 85th percentile (normal weight), 85th ≤ BMI < 95th percentile (overweight), and BMI ≥ 95th percentile (obese)

#### Accuracy, bias, and precision by speed

A catalog of validity indices of accuracy (MAPE), bias (MPE), and precision (SD and CoV) for step counting wearable technologies at different speeds, wear locations, and age groups is provided in Additional file 5. Additionally, MAPE, MPE, and their corresponding SD values across the full range of speeds are visually presented in Additional file 6 for the whole sample and by age groups. According to the catalog and over the range of normal speeds (4.0–6.4 km/h), the Actical displayed the highest accuracy (MAPE = 1%), followed by the NL-1000 (3%), the activPAL, SW-200 and StepWatch (4%), and the waist-worn ActiGraph GT3X+ (7%). On the other hand, the SenseWear reported the lowest accuracy across normal speeds (MAPE = 62%), followed by the wrist-worn ActiGraph GT3X+ (41%). When considering the whole range of slow, normal and fast speeds, the StepWatch displayed the highest accuracy (MAPE = 9%), followed by the activPAL (12%) and the Actical (19%). In contrast, the SenseWear reported the lowest accuracy (MAPE = 73%), followed by the wrist- and waist-worn ActiGraph GT3X+ (51 and 34% respectively), the NL-1000 (23%), and the SW-200 (22%). On average, all wearable technologies displayed the lowest accuracy across slow walking speeds (MAPE = 50.1 ± 35.5%), and the highest accuracy across normal speeds (MAPE = 15.9 ± 21.7%). Only the StepWatch reported the lowest accuracy across fast speeds (22%). The correlation coefficients representing the strength of the relationship between directly observed steps and steps derived from each wearable technology are reported in Additional file 7.

#### Accuracy, bias, and precision by wear location

Validity indices averaged across wear locations for each speed bout are presented in Table 2. Across normal speeds, the waist (MAPE = 4%), thigh (4%) and ankle (5%) locations displayed higher accuracy than the wrist location (23%). Across the whole range of speeds, the ankle and the thigh locations showed the highest accuracy (MAPE = 9 and 11%, respectively), while the wrist and waist locations showed the lowest accuracy (MAPE = 30 and 24%, respectively). Mean precision (correlation) values (and 95% CIs) are presented in Supplementary Figure 2, Additional file 8 for the relationship between directly observed steps and steps averaged across each specific wear location-based devices.

**Table 2:** Validity indices (accuracy, bias, and precision) averaged across wear location for each speed bout

	Treadmill speed, km/h (mph)									
	Slow				Normal				Fast	
	0.8 (0.5)	1.6 (1.0)	2.4 (1.5)	3.2 (2.0)	4.0 (2.5)	4.8 (3.0)	5.6 (3.5)	6.4 (4.0)	7.2 (4.5)	8.0 (5.0)
***Waist***										
**MAPE ± SD**	92.4 ± 11.2	74.1 ± 25.2	40.7 ± 26.8	16.6 ± 18.3	5.9 ± 12.1	4.1 ± 11.6	2.7 ± 8.9	3.1 ± 9.1	2.6 ± 8.6	2.6 ± 6.1
**MPE ± SD**	− 92.4 ± 11.2	−74.1 ± 25.3	− 39.7 ± 28.2	− 15.3 ± 19.4	−5.0 ± 12.5	−2.6 ± 12.0	−1.7 ± 9.2	−1.7 ± 9.4	−0.9 ± 8.9	1.2 ± 6.6
***Wrist/Arm***										
**MAPE ± SD**	93.8 ± 9.4	83.6 ± 19.0	68.5 ± 28.1	56.2 ± 34.3	52.4 ± 37.1	50.1 ± 36.8	51.2 ± 36.1	52.2 ± 33.8	54.9 ± 32.0	53.2 ± 28.7
**MPE ± SD**	− 93.8 ± 9.4	− 83.6 ± 19.0	− 68.4 ± 28.3	− 56.1 ± 34.5	−52.3 ± 37.2	−49.7 ± 37.3	−51.1 ± 36.2	−52.0 ± 34.1	− 54.6 ± 32.5	− 51.6 ± 31.5
***Thigh***										
**MAPE ± SD**	60.2 ± 28.2	10.9 ± 14.7	3.2 ± 8.7	3.5 ± 9.0	3.2 ± 8.9	3.3 ± 9.2	3.7 ± 9.9	6.0 ± 14.7	9.8 ± 23.1	11.1 ± 24.7
**MPE ± SD**	− 59.5 ± 29.7	−8.9 ± 16.0	−1.6 ± 9.1	−1.2 ± 9.5	−1.8 ± 9.3	−1.7 ± 9.7	− 2.1 ± 10.4	−5.0 ± 15.1	−8.5 ± 23.6	−7.2 ± 26.2
***Ankle***										
**MAPE ± SD**	14.9 ± 19.8	4.4 ± 13.1	3.4 ± 13.2	3.6 ± 13.8	3.1 ± 13.4	2.9 ± 10.4	4.1 ± 12.0	8.7 ± 15.7	20.7 ± 18.0	23.5 ± 7.9
**MPE ± SD**	− 14.6 ± 20.0	−0.6 ± 13.8	−1.5 ± 13.5	−1.3 ± 14.2	−1.8 ± 13.6	−1.1 ± 10.8	−3.0 ± 12.4	−8.0 ± 16.1	−20.4 ± 18.4	−23.5 ± 7.9

All mean absolute percentage error (MAPE) and mean percentage error (MPE) values are presented as mean percentage ± standard deviation (SD). Waist devices: Actical, ActiGraph GT3X+, NL-1000, Digi-Walker SW-200. Non-dominant wrist device: ActiGraph GT3X+; Arm device: SenseWear. Thigh device: activPAL. Ankle device: StepWatch. MAPE and SD values closer to 0% indicate higher accuracy and precision, respectively. MPE values closer to 0% indicate lower bias and negative values mean undercounting

#### Accuracy, bias, and precision by age group

Validity indices averaged across age groups for each speed bout are presented in Table 3. Accuracy ranged from MAPE = 81.2% (adolescents at 0.8 km/h) to 14.4% (young adults at 5.6 km/h). The wearable devices’ accuracy across normal speed bouts was similar among age groups (children’s MAPE = 17%, adolescents = 16%, young adults = 15%). Across all speed levels, MAPE values were also similar among age groups (children = 32.1%, adolescents = 30.4%, young adults = 29.6%). Mean precision (correlation) values (and 95% CIs) are represented in Supplementary Figure 3, Additional file 8 for the relationship between steps averaged across each age group and directly observed steps.

**Table 3:** Validity indices (accuracy, bias, and precision) averaged across all wearable technologies and presented by age group

	Treadmill speed, km/h (mph)									
	Slow				Normal				Fast	
	0.8 (0.5)	1.6 (1.0)	2.4 (1.5)	3.2 (2.0)	4.0 (2.5)	4.8 (3.0)	5.6 (3.5)	6.4 (4.0)	7.2 (4.5)	8.0 (5.0)
***Children (6***–***12 years)***										
**MAPE ± SD**	76.7 ± 30.0	58.2 ± 36.2	38.0 ± 32.6	24.1 ± 30.1	17.3 ± 29.3	16.0 ± 28.3	16.3 ± 28.9	18.5 ± 28.1	24.1 ± 30.2	–
**MPE ± SD**	−76.7 ± 30.0	− 57.6 ± 37.1	−37.6 ± 33.1	−22.8 ± 31.0	− 16.7 ± 29.6	− 14.3 ± 29.2	−15.3 ± 29.4	−17.5 ± 28.8	−22.4 ± 31.5	–
***Adolescents (13***–***17 years)***										
**MAPE ± SD**	81.2 ± 30.0	61.7 ± 37.7	39.4 ± 33.6	23.6 ± 29.9	17.0 ± 30.0	14.9 ± 29.1	14.6 ± 28.7	16.0 ± 28.8	18.6 ± 29.3	16.5 ± 25.1
**MPE ± SD**	−80.9 ± 31.2	− 60.8 ± 39.1	−38.2 ± 35.0	−22.3 ± 30.9	−16.0 ± 30.5	−14.0 ± 29.6	−13.5 ± 29.2	−15.1 ± 29.3	−17.6 ± 29.9	−15.4 ± 25.8
***Young adults (18***–***20 years)***										
**MAPE ± SD**	80.6 ± 31.9	60.6 ± 38.7	36.9 ± 35.5	21.0 ± 28.8	15.8 ± 29.4	14.9 ± 29.5	14.4 ± 28.7	14.9 ± 28.4	16.2 ± 28.9	20.4 ± 28.4
**MPE ± SD**	− 80.6 ± 32.1	− 59.7 ± 40.0	−35.4 ± 37.1	−19.6 ± 29.8	−14.8 ± 29.9	−14.8 ± 29.9	−13.8 ± 29.1	−13.9 ± 28.9	−15.3 ± 29.4	−16.6 ± 30.9

All mean absolute percentage error (MAPE) and mean percentage error (MPE) values are presented as mean percentage ± standard deviation (SD). MAPE and MPE values were calculated for all wearable technologies (Actical, ActiGraph GT3X+ [waist], ActiGraph GT3X+ [non-dominant wrist], activPAL, Digi-Walker SW-200, NL-1000, SenseWear, and StepWatch) when compared to the directly observed steps. MAPE and SD values closer to 0% indicate higher accuracy and precision, respectively. MPE values closer to 0% indicate lower bias and negative values mean undercounting

### Inferential analyses

#### Effect of speed on accuracy, bias, and precision

**Fig. 1:**
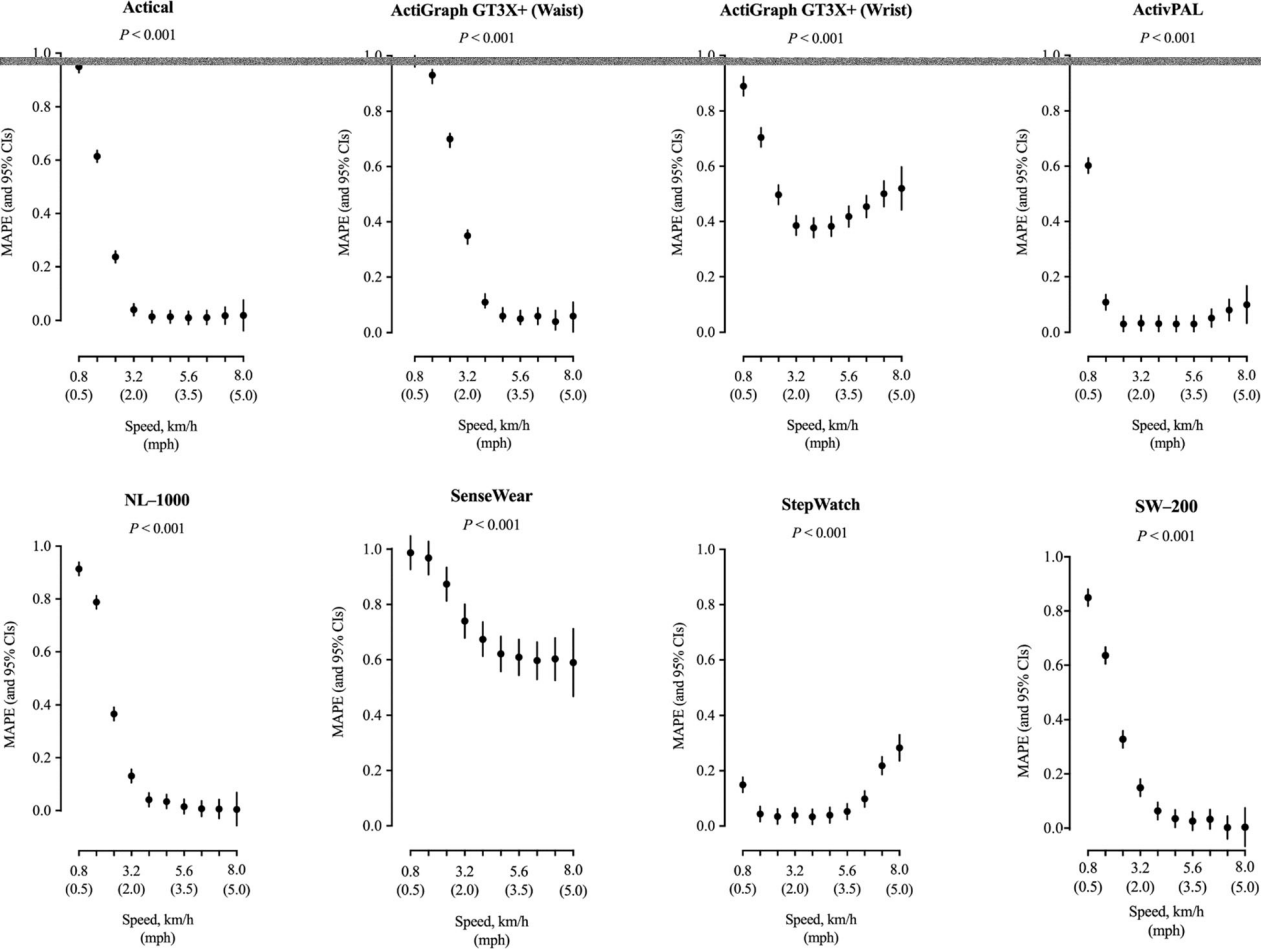
Effect of speed on overall accuracy (mean absolute percentage error, MAPE) of wearable technology’s step counting ability. Participants walked on a treadmill for 5-min bouts beginning at 0.8 km/h (0.5 mph) and increasing in 0.8 km/h (0.5 mph). MAPE and corresponding 95% confidence intervals (CIs) respective to each technology are plotted across speed bouts. Each point represents grouped averages of MAPE values, with 95% CIs estimated using mixed effect models and extending above and below that point estimate. MAPE values closer to 0 are indicative of greater accuracy. Further, where 95% CIs do not overlap, there are significant differences between speed bouts. Likelihood ratio test *P* value is reported for the effect of all speeds on MAPE for each specific device

The regression models built for each of the eight tested wearable technologies in comparison with the directly observed steps indicated that there was an overall significant effect of speed (*P* < 0.001) on accuracy (Fig. 1), and that this significance was generally driven by a reduced accuracy (i.e., increased MAPE) at slow speeds (0.8–3.2 km/h). For example, MAPE values were significantly greater at 0.8 km/h compared to 1.61 km/h for all devices (except for SenseWear). Across normal and fast walking speeds (4.0–8.0 km/h), there were overall no significant differences in accuracy (*P* > 0.05), with the exception of waist-worn ActiGraph GT3X+ (MAPE, 95% CI = 0.06, 0.04–0.19 at 4.8 km/h and 0.05, 0.03–0.08 at 5.6 km/h), and StepWatch (MAPE, 95% CI = 0.22, 0.19–0.25 at 7.2 km/h and 0.10, 0.07–0.13 at 6.4 km/h). We observed similar findings for bias since speed had an overall significant effect (*P* < 0.001) on MPE values across all wearable technologies (see Supplementary Figure 1, Additional file 9), mainly during slower walking speeds.

The regression modeling indicated that speed did not have an effect (*P* = 0.235) on the correlation between irectly observed steps and steps derived from wearable technology (see Supplementary Figure 1, Additional file 8).

#### Effect of wear location on accuracy, bias, and precision

**Fig. 2:**
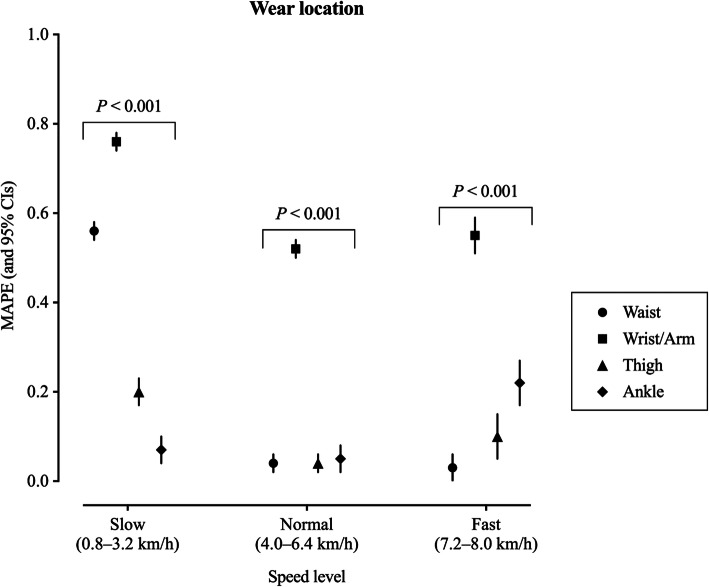
Effect of wear location on overall accuracy (mean absolute percentage error, MAPE) of wearable technologies’ step counting ability. MAPE and corresponding 95% confidence intervals (CIs; estimated using mixed effect models) of each wear location are presented at slow, normal, and fast walking speed levels. Slow speed bouts: 0.8, 1.6, 2.4, 3.2 km/h (0.5, 1.0, 1.5, 2.0 mph); normal speed bouts: 4.0, 4.8, 5.6, 6.4 km/h (2.5, 3.0, 3.5, 4.0 mph); fast speed bouts: 7.2, 8.0 km/h (4.5, 5.0 mph). MAPE values were averaged across devices respective to each wear location for slow, normal, and fast walking speeds. MAPE values closer to 0 are indicative of greater accuracy. Further, where 95% CIs do not overlap, there are significant differences between locations. Likelihood ratio test *P* value is reported for the effect of wear location on MAPE for each specific speed level

The regression models indicated that wear location had a significant effect on accuracy associated with measuring steps (*P* < 0.001) across slow, normal, and fast walking speeds (Fig. 2). Across slow and fast walking speeds, all locations showed significantly different MAPE (95% CI) among them with highest MAPE values (lowest accuracy) being 0.76 (0.74–0.78) and 0.55 (0.51–0.58) reported from wrist/arm for slow and fast speeds, respectively, and lowest MAPE values (highest accuracy) being 0.07 (0.04–0.10) and 0.03 (0.00–0.05) reported from ankle and waist locations for slow and fast speeds, respectively. Across normal walking speeds, waist, thigh, and ankle accuracy was not significantly different from each other (overlapping 95% CIs ranging from 0.02 to 0.07), with wrist/arm showing a significantly different accuracy (averaged MAPE = 0.52, 95% CIs: 0.50–0.53) with respect to the other locations. Similarly, the regression modeling also indicated that there was a significant effect of wear location on bias (MPE) during slow, normal, and fast walking speeds (*P* < 0.001) (see Supplementary Figure 2, Additional file 9).

Wear location did not have an overall significant effect on the correlation between directly observed steps and those derived from wearable technologies (*P* > 0.05) (see Supplementary Figure 2, Additional file 8). The only significant difference observed was between waist and wrist/arm, as the 95% CIs did not overlap (0.74–1.00 for waist, and 0.34–0.72 for wrist/arm).

#### Effect of age on accuracy, bias, and precision

**Fig. 3:**
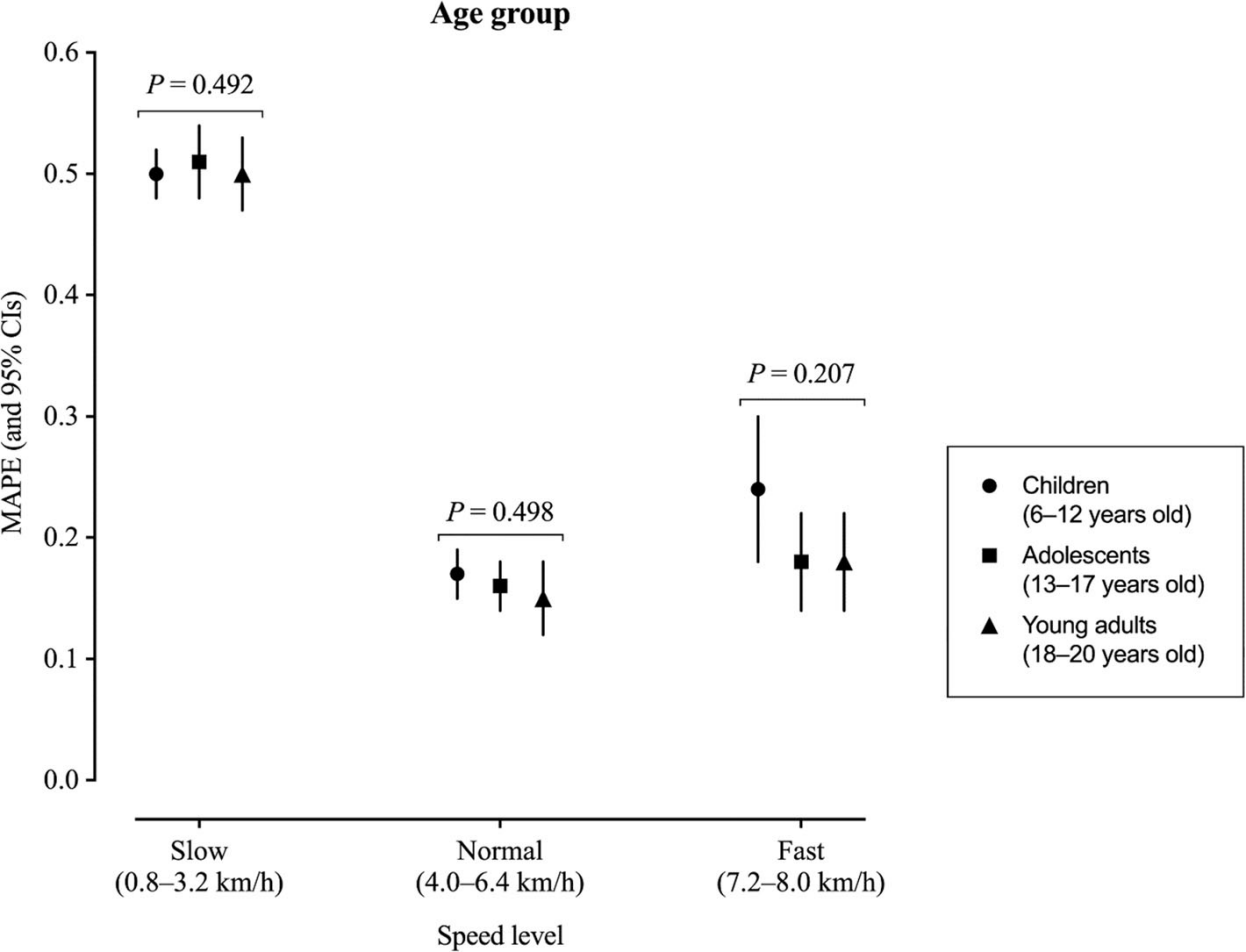
Effect of age on overall accuracy (mean absolute percentage error, MAPE) of wearable technologies’ step counting ability. MAPE and corresponding 95% confidence intervals (CIs; estimated using mixed effect models) of each age group are presented at slow, normal, and fast walking speed levels. Slow speed bouts: 0.8, 1.6, 2.4, 3.2 km/h (0.5, 1.0, 1.5, 2.0 mph); normal speed bouts: 4.0, 4.8, 5.6, 6.4 km/h (2.5, 3.0, 3.5, 4.0 mph); fast speed bouts: 7.2, 8.0 km/h (4.5, 5.0 mph). MAPE values were averaged across devices respective to each age group for slow, normal, and fast walking speeds. MAPE values closer to 0 indicate greater accuracy. Further, where 95% CIs do not overlap, there are significant differences between locations. Likelihood ratio test P value is reported for the effect of age on MAPE for each specific speed level

The regression models indicated that age did not have a significant effect on the step counting accuracy (Fig. 3), bias (see Supplementary Figure 3, Additional file 9) and precision (see Supplementary Figure 3, Additional file 8) of wearable technologies at slow, normal, or fast walking speeds or across speed levels (all *P* ≥ 0.05).

## Discussion

The highly detailed and standardized digital catalog assembled herein clearly demonstrates the effect that speed and wear location have on step counting performance of wearable technologies with regards to accuracy and bias, with no apparent effect on precision. In contrast, age had no effect on any validity index evaluated in this sample ranging from children to young adults. Users can search the catalog (Additional file 5) for any validity index (i.e., MAPE, MPE, SD, CoV) as well as tailor the search as needed using a filter for any of the factors provided (i.e., speed, location, age, or specific devices). The catalog also includes additional worksheets that display the validity indices for each tested device separately and can be used by researchers to inform wearable technology selection and to compare data collected by different devices. This catalog of validity indices extends the scoping review recently published that called for a harmonization of wearable technologies validation [15]. Wearable technologies manufacturers also now have a set of evidence-based validity parameters that can be referenced to support development of new devices in an effort to assure standardized performance.

### Descriptive findings for accuracy, bias, and precision

Five of the eight tested wearable technologies (i.e., Actical, waist-worn ActiGraph GT3X+, activPAL, StepWatch, and SW-200) performed at < 5% MAPE over the range of normal walking speeds. Although the CTA previously suggested that wearable technologies can be expected to perform at an accuracy level of < 10% MAPE [18], the empirical basis for this assertion was not clear. In contrast, the reference values produced herein can actually provide such data to inform more evidence-based thresholds as suggested in previous review [15].

When selecting appropriate wearable technologies, researchers should first consider the population of interest. For example, if the population of interest typically walks at slow walking speeds, the best technology for this speed appears to be different than what it would be for those more capable of normal or fast walking. Specifically, at slow walking, the best devices based on accuracy were: StepWatch (MAPE = 6.6%), activPAL (19.4%), Actical (46.0%), SW-200 (49.0%), NL-1000 (55.0%), wrist-worn ActiGraph GT3X+ (61.8%), waist-worn ActiGraph GT3X+ (73.8%), and SenseWear (89.2%). While the CTA [18] recommended threshold of MAPE < 10% is intended for normal walking speeds (4.0–6.4 km/h), the StepWatch’s MAPE fell below this threshold also during slow walking speeds. Of note, this rank order for slow walking speeds was the same for MPE. Under normal walking speeds, best devices based on accuracy were: Actical (MAPE = 1.4%), NL-1000 (2.9%), SW-200 (4.0%), activPAL (4.1%), StepWatch (4.7%), waist-worn ActiGraph GT3X+ (7.4%), wrist-worn ActiGraph GT3X+ (40.6%), and the SenseWear (62.3%). Actical, waist-worn ActiGraph GT3X+, activPAL, StepWatch, and SW-200 actually performed at a more rigorous level of < 5% MAPE across this normal speed range. Again, this order at normal walking speeds did not change with respect to MPE. In regards to fast walking, the best devices based on accuracy were: SW-200 (MAPE = 1.6%), NL-1000 (1.7%), Actical (2.2%), waist-worn ActiGraph GT3X+ (5.0%), ActivPAL (10.5%), StepWatch (22.1%), wrist-worn ActiGraph GT3X+ (47.9%), and Sensewear (60.2%). The rank order did change slightly with respect to MPE, where the NL-1000 displayed the least amount of error (0.5%) followed by the SW-200 (1.0%). Guided by these findings we can affirm that the cost of the wearable technology does not necessarily guarantee a degree of accuracy, bias and precision, as demonstrated here by a ~ $20 pedometer (e.g., SW-200) outperforming several ~$200+ devices (e.g., Actigraph GT3X+).

### Effect of speed on accuracy, bias, and precision

In the present study, speed had a significant effect on accuracy and bias. This effect was primarily driven by greater error evident at slower speeds, with better accuracy during normal walking speeds. Although not restricted to youth samples, Bassett et al. [8] suggested that wearable technologies only record 50–75% of the actual number of steps taken at walking speeds ≤1.6 km/h (1.0 mph), i.e., 50–25% MAPE. The findings herein agree with this assertion, apparent from the finding that grouped mean values across wearable technologies at speeds ≤1.6 km/h missed 30.5% of actual steps taken (within that 50–25% MAPE range). Given these findings, manufacturers of wearable technologies should thoroughly consider measurement’s trade-off between sensitivity and specificity to avoid miscategorization of low force accelerations that are true steps [24].

Overall, there were no significant differences in accuracy or bias for any of the tested wearable technologies across increasing speeds during normal and fast walking. An improvement of accuracy and bias was observed from slow to normal and fast speeds. For instance, while MAPE averaged across waist, thigh and ankle-worn devices was 42% across slow walking speed bouts, the MAPE across normal walking speed bouts was 4%. This trend of improved MAPE values with increased speed (≥ 4.0 km/h [2.5 mph]) is consistent with previous literature [9, 19, 25, 27, 31–33]. For example, Trapp et al. [27] reported improving MAPE from 44.1 to 8.9% from slow (2.4 km/h [1.5 mph]) to fast (5.4 km/h [3.4 mph]) walking speed for waist-worn devices.

Although accuracy and bias were significantly influenced by speed, precision (as represented by correlation coefficients) was not similarly affected. A methodological explanation for this could be the apparent difference in power in the regression analyses. While the regression models for the correlation coefficients were averaged across the eight wearable technologies for each of the speed categories of slow, normal and fast (in order to provide a more robust correlation for a given coefficient), the regression models for MAPE and MPE considered more data points, as values were averaged across the total sample for each speed bout. This decrease in power related to precision analyses also increased the width of the associated 95% CIs, which in turn increased the likelihood of apparent overlap between speed levels, an indication of no significant differences. Further, MAPE, MPE, and correlation coefficients are different by nature. While correlation coefficients are unitless values that indicate the strength of the linear relationship between directly observed steps and steps derived from wearable technology, MAPE and MPE are scaled measures (%) that define the amount of error of a device when counting steps. The strength of the linear relationship is not representative of the magnitude of error in respect to directly observed steps. To be clear, a wearable technology could consistently (i.e., precisely) undercount the same number of steps respective to directly observed steps, and therefore, display a high correlation coefficient, despite consistent error (more apparent from inexact MAPE and MPE values).

### Effect of wear location on accuracy, bias, and precision

Wear location also had a significant effect on the accuracy and bias of wearable technologies step counting. Consistently, wearable technologies worn at the wrist/arm performed significantly worse than those worn on the waist, thigh, or ankle in terms of accuracy and bias, regardless of the speed. During normal walking speeds, waist, thigh, and ankle-worn devices were all comparable, and within 5% error for both MAPE and MPE values. The better accuracy and bias values reported by these locations may be a consequence of a higher sensitivity to accelerations and gravitational forces occurring with each step taken during ambulation. For example, devices worn at the thigh and ankle undergo greater displacement during ambulation due to their proximity to the foot [34]. Specifically, Sandroff et al. [34] reported a 0.03% MAPE at 1.6 km/h (1.0 mph) for the thigh-worn StepWatch in adults with multiple sclerosis. Although we observed slightly less accurate measures of the StepWatch at the same speed for the sample herein (MAPE = 4.4%), this device was still the most accurate wearable technology tested at this speed. As reported in Tudor-Locke et al. [33], another explanation of the differences between wrist/arm and other wear locations is that acceleration/force signals at the wrist/arm are not sufficient to be effectively and consistently detected. In Tudor-Locke et al. study in adults [33], wrist-worn devices produced outputs that were significantly different from directly observed steps at speeds ranging from 0.8 to 11.3 km/h, while waist-worn devices displayed no significant differences at speeds > 3.2 km/h. In the present study in children and young adults, we observed similar findings during normal walking speeds as waist, thigh, and ankle MAPE values were not significantly different from each other, while wrist/arm showed a significantly different MAPE with respect to the other locations.

At fast walking speeds, accuracy and bias appeared to worsen for the wrist/arm, thigh, and ankle-worn devices, while that for the waist-worn devices improved. From normal to fast walking, all four of the wrist/arm, thigh, and ankle-worn devices displayed increased MAPE (3.0 to 21.0% difference from normal to fast speeds), indicating decreased performance at higher speeds. These findings are in line with previous studies with, for example, the study of Aminian et al. [22] showing a steps’ underestimation of − 8% of a thigh device during fast walking similar to the − 8.5% presented in our study. On the other hand, the improvement of accuracy from normal to fast speeds of the waist-worn devices showed in our study does not correspond with findings from previous studies [21, 22]. Thus, in the mentioned studies [21, 22], the waist-worn devices displayed increasing error from normal to fast walking speeds. These differences in accuracy or bias for waist-worn devices between our study and previous ones may be a result of the use of different brands/models of devices.

As with speed, there was no effect of wear location on precision as represented by correlation coefficients. Again, this inconsistency of significance across validity indices is due to a reduced power in the regression models respective to each index, as described previously. Further, there were only two devices worn at the wrist/arm, one at the thigh, and one at the ankle which further reduces the amount of data points included in regression models for correlation coefficients, and in turn produces wider 95% CIs, indicative of no significant differences.

### Effect of age on accuracy, bias, and precision

Age showed no effect on any of the validity indices at slow, normal, or fast walking and for all bouts in this sample ranging from children as young as 6 years of age to young adults aged 20 years of age. No previous studies have directly analyzed the effect of age on performance of wearable technologies which hampers direct comparison with our findings. MAPE and MPE values were ≤ 8% different between age groups, and respective to correlation coefficients, values were ≤ *r* = 0.06 different among children, adolescents and young adults. One explanation for the non-apparent effect of age is that beyond 6 years of age, there are no notable differences in walking patterns compared to those associated with adults [35]. According to our findings, PA studies and interventions designed to capture steps in young people do not need to consider age groups in terms of measurement with step counting wearable technologies. However, since postural and locomotor control may differ in very young children [35], future studies should seek to include even younger and older samples.

### Strengths and limitations

Several strengths of the original study design underpinning this secondary analysis must be acknowledged, including the use of direct observation of step counting and video back up as criterion measure, as recommended by the CTA [18]. Additionally, the treadmill protocol covered a broad range of walking speeds from 0.8 km/h (0.5 mph) to 8.0 km/h (5.0 mph) which extends beyond the data collected in previous studies [21–27]. Thirdly, this is the first study in a young population to report all three validity indices in a large sample size (*N* = 117), evenly distributed by age year. There are several limitations that must also be acknowledged. This analysis included the evaluation of only eight step counting wearable technologies. At the wrist, arm, thigh and ankle, only a single device was evaluated, limiting generalization to other devices that can also be worn at these locations. For example, we used a research-grade ActiGraph GT3X+ as the only device at the wrist, while we used four different devices on the waist. Regardless, this secondary analysis from the primary CADENCE-Kids study [28] is consistent with the evidence shown by a recent review on the topic [17] and lays the foundation for a catalog that future research should be able to add to as emerging devices (and new algorithms for data processing) are evaluated using similar protocols. While this was a more extensive array of devices than previously evaluated [21–27], there are still many other wearable technologies available that were not included in this original data set. Some of the originally tested wearable technologies are also now obsolete (e.g., SenseWear), however, publication of these specific validity-related values is still important to enable robust comparisons between different types of past, present, and future wearable technologies. Another limitation is that the size of the available analytical sample diminished as speed increased in the original study. Regarding classifying speed as low, normal and fast, we followed the definition of the normal speed range made by the CTA [18], and then defined low as less than that range and fast as higher than that range. While we acknowledge that this definition is most relevant to adults, there are no separate standards for step counting accuracy set in children yet. Lastly, it is important to note that the step-based validation of these wearable technologies on a treadmill may not be generalizable to the free-living condition. The sporadic, and non-uniform patterns of children’s movement may further limit the generalizability of these step count data when quantifying free-living step-based PA behavior [36].

## Conclusion

The results reported and discussed herein are an important first step to standardizing the validation of step counting wearable technologies. This effort provides comprehensive validity indices for a variety of step counting wearable technologies evaluated during treadmill walking in young people 6–20 years of age. Cataloging expected validation indices for step counting wearable technologies in young samples is important to inform and facilitate empirically-based reference values needed to evaluate accuracy, bias, and precision of new devices. Speed and wear location had a significant effect on the accuracy and bias of wearable technologies step counting ability, but not the precision. However, age did not influence wearable technology performance with regards to any of these three validity indices. Future research should continue to rigorously validate new wearable technologies as they are developed, and also extend this standardized laboratory-based evaluation to the free-living environment.

## Supplementary Information

**Additional file 1.** Table displaying step counting treadmill validation studies in youth.

**Additional file 2.** Tabular and visual presentations of the eight wearable technologies worn by the participants.

**Additional file 3.** Spreadsheets displaying the final analytical data set and the corresponding data dictionary.

**Additional file 4.** Tables displaying sample sizes, and number of steps derived by each treadmill speed for all sample and by age groups.

**Additional file 5.** Catalog of validity indices for step counting wearable technologies at different speeds, wear locations, and age groups.

**Additional file 6.** Figures for descriptive statistics representing accuracy, precision and bias for each wearable technology.

**Additional file 7 **Tabular and graphical representations of correlation coefficients (*r*) of the relationship between directly observed steps and steps derived from wearable technologies.

**Additional file 8.** Figures of the effect of speed, wear location, and age on the overall precision of wearable technologies step counting ability.

**Additional file 9.** Figures of the effect of speed, wear location, and age on bias of wearable technologies step counting ability.

## Data Availability

All data generated or analyzed during this study are included in this article and its additional files.

## Abbreviations

BMI: Body mass index
CI: Confidence interval
CoV: Coefficient of variation
CTA: Consumer Technology Association
km/h: Kilometers per hour
MAPE: Mean absolute percentage error
mph: Miles per hour
MPE: Mean percentage error
PA: Physical activity
RMSE: Root mean squared error
SD: Standard deviation
