# Supplementary material for: A catalog of validity indices for step counting wearable technologies during treadmill walking: the CADENCE-Kids study
Source: Int J Behav Nutr Phys Act. 2021 Jul 16;18:97. doi: 10.1186/s12966-021-01167-y (PMC8283935; doi:10.1186/s12966-021-01167-y)
Supplement: Supplementary file 1 — Additional file 1. Table displaying step counting treadmill validation studies in youth. [file 12966_2021_1167_MOESM1_ESM.pdf]

Suppl Table 1 Step counting treadmill validation studies among wearable technologies in youth

Reference	Sample	Protocol (duration and speeds)	Wearable Technologies	Accuracy	Precision	Bias
Pedometer Studies						
Beets, Patton and Edwards 2004	20 children (50% females), 5 to 11 years	2-min: 40, 54, 67, 80, and 94 m/min	Walk4Life 2505 (WL), Digiwalker SW-200 (DW200), Sun TrekLINQ (SUN) and Digiwalker SW-701 (DW701)	MAPE:<br><br>Only visualized in graph	ICC:<br><br>WL: 0.516-0.992<br><br>DW200: 0.727-0.998<br><br>SUN: 0.330-0.971<br><br>DW701: 0.722-0.993	Not reported
Trapp et al. 2012	45 children (51.1% females), age 10.67 ± 0.77 years	3-min: 40, 67, 91 m/min	Accuspllit AH120, DigiWalker SW-700	MAPE:<br><br>AH120 = 46.9-8.6%<br><br>SW-700 = 44.1-8.9%	Not reported	Bland-Altman Plots

Ramírez-Marrero et al. 2002	31 children (45.2% females) 7 to 12 years	2 min at 59, 70, 91 m/min	Digiwalker SW-200 (hip; belt), Digiwalker SW-200 (midline; pouch)	MAPE:<br>Hip = 1-15%<br>Midline = 2-5%	Not reported	Not reported
Mitre et al. 2009	27 children (51.9% females) 8 to 12 years	5-min: 13.4, 26.8, 40.2, 53.6 m/min	Omron HJ-105, DigiWalker SW-200	Not reported	Not reported	MPE:<br>Omron HJ-105 and DigiWalker SW-200: 100-60%<br>Bland-Altman Plots

Dueker, Gauderman, and McConnell, 2012	17 children (58.8% females), aged 10 to 17 years	5-min: 53.6, 80.5, 107.3, 134.1 m/min	SportBrain iStep X1, Digiwalker SW-701	MAPE	Not reported	MPE:<br><br>iStep X1= -1.3 to -3.9%<br><br>SW-701= -19.3 to -4.3%
Aminian and Hinckson, 2012	25 children (68% females) 9.9 ± 0.3 years	2-min: 50, 66, 93, 133 m/min	Yamax DigiWalker SW-200 and NL-2000	Not reported	Not reported	MPE:<br>SW-200:<br><br>-4% to 1%<br><br>NL-2000:<br><br>-11% to 1%
Rosenkranz, Rosenkranz, and Weber, 2011	19 children (36.8% female) aged 7–11 years	3-min 40.2, 80.5, 120.7, 161 m/min	Digiwalker SW-200	Not reported	Not reported	MPE: -4%<br><br>(not specified by speeds)

Accelerometer Studies

Aminian and Hinckson, 2012	25 children (68% females) 9.9 ± 0.3 years of age	2/min: 50, 66, 93, 133 m/min	ActivPAL	Not reported	ICC:<br>ActivPAL: 0.21-1.00	Not reported
Rosenkranz, Rosenkranz, and Weber, 2011	19 children (36.8% female) aged 7–11years	3-min: 40.2, 80.5, 120.7, 161 m/min	Actical (waist), Actical (ankle)	Not reported	ICC:<br>Waist: 0.927<br>Ankle: 0.854 (not specified by speeds)	MPE:<br>Waist: -11%<br>Ankle: -17%<br>(not specified by speeds)

ICC = Intra-class coefficient; MAPE = Mean absolute percentage error; MPE = Mean percentage error
