# Supplementary material for: A catalog of validity indices for step counting wearable technologies during treadmill walking: the CADENCE-Kids study
Source: Int J Behav Nutr Phys Act. 2021 Jul 16;18:97. doi: 10.1186/s12966-021-01167-y (PMC8283935; doi:10.1186/s12966-021-01167-y)
Supplement: Supplementary file 2 — Additional file 2. Tabular and visual presentations of the eight wearable technologies worn by the participants. [file 12966_2021_1167_MOESM2_ESM.docx]

**Supplementary Table 1, Additional File 2**. Relation of wearable technologies used in the present study

| **Wearable** | **Location** | **Digital Display** | **Data Extraction Software** | **Initial Step Data Resolution (Epoch)** | **Device Settings and Initialization Information** |
| --- | --- | --- | --- | --- | --- |
| Actical | Left waist | No | Actical® (v. 2.12.0002) | 15 s | - Epoch length: 0.25. - “*Record Steps?*” box checked. |
| ActiGraph GT3X+ | Right waist | No | ActiLife (v. 6.11.8) | 1 s | - Sampling rate: 80 Hz. - LED flashes not checked. - “*Idle sleep mode*” disabled. - “*Low Frequency Extension*” disabled. - *“By default”* algorithm for step counting analysis. |
| ActiGraph GT3X+ | Non-dominant wrist | No | ActiLife (v. 6.11.8) | 1 s | - Sampling rate: 80 Hz. - LED flashes not checked. - “*Idle sleep mode*” disabled. - “*Low Frequency Extension*” disabled. - *“By default”* algorithm for step counting analysis. |
| activPAL | Right thigh | No | activPAL™ process and presentation (v. 6.4.1) | 15 s | - *“Future Time”* selected for Start condition. - “*Record for 1 day*” selected. |
| New Lifestyles NL-1000 | Right waist | Yes | N/A | 5 min | - Mode button pushed to display “*STEPS*”. |
| SenseWear Armband | Right arm | No | SenseWear® Professional (v. 7.0) | 60 s | - Under “*Armband Parameters*” tab, “*Apply*” button pushed to configure armband. |
| StepWatch | Right ankle | Yes | StepWatch™ Analysis Software (v. 3.1) | 3s | - Recording Interval of “*3 seconds*” selected. - “*Set to Max*” button selected. |
| SW-200 | Left waist | Yes | N/A | 5 min | - Press “*RESET*” button to clear the currently stored “*STEP*” value. |

Devices with “N/A” listed for Data Extraction Software were standalone devices which did not require interfacing with a computer for data extraction – data read directly from available visual displays at the end of each 5-minute stage

**
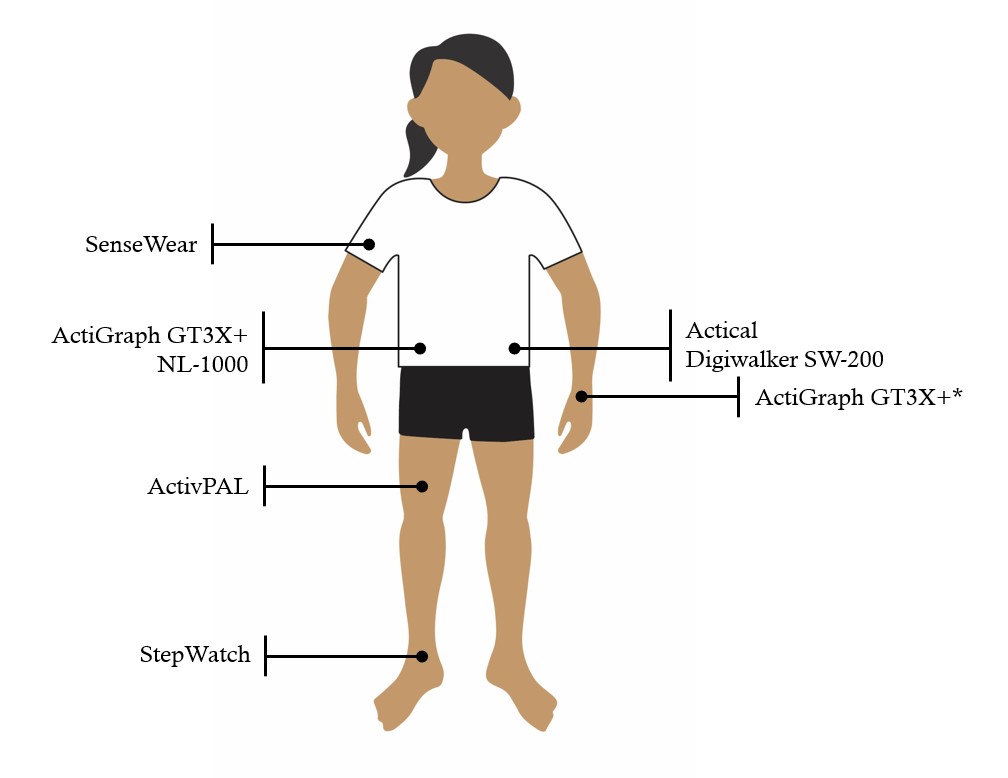
**

**Supplementary Figure 1, Additional File 2**. Visual representation of all wearable technologies and their locations. *ActiGraph GT3X+ located on the wrist was placed on the non-dominant one.
