# Supplementary material for: A catalog of validity indices for step counting wearable technologies during treadmill walking: the CADENCE-Kids study
Source: Int J Behav Nutr Phys Act. 2021 Jul 16;18:97. doi: 10.1186/s12966-021-01167-y (PMC8283935; doi:10.1186/s12966-021-01167-y)
Supplement: Supplementary file 4 — Additional file 4. Tables displaying sample sizes, and number of steps derived by each treadmill speed for all sample and by age groups. [file 12966_2021_1167_MOESM4_ESM.pdf]

Supplementary Table 1, Additional File 4. **S**ample sizes (*N*) and number of steps derived from direct observation and each wearable technology by each treadmill speed for all sample (6–20 years old)

	**Treadmill speed, km/h (mph)**									
	**Slow**				**Normal**				**Fast**	
	**0.8 (0.5)**	**1.6 (1.0)**	**2.4 (1.5)**	**3.2 (2.0)**	**4.0 (2.5)**	**4.8 (3.0)**	**5.6 (3.5)**	**6.4 (4.0)**	**7.2 (4.5)**	**8.0 (5.0)**
N	*117*	*117*	*115*	*113*	*110*	*104*	*93*	*81*	*53*	*16*
**Direct observation**	321.6 ± 90.9	396.7 ± 83.1	454.1 ± 71.3	503.5 ± 59.1	553.5 ± 51.5	592.1 ± 52.1	634.1 ± 56.6	684.8 ± 72.4	746.1 ± 70.6	762.1 ± 40.2
Min–Max	161–603	255–617	338–709	396–736	472–706	508–816	535–850	577–895	611–870	693–858
**Actical**	20.4 ± 49.5	147 ± 95.5	350.3 ± 89.1	490.4 ± 61.3	550.7 ± 53	592.4 ± 53.3	635 ± 57.7	687.1 ± 77.5	748 ± 76.5	776.7 ± 40.6
Min–Max	0–377	0–367	123–555	230–682	343–690	482–840	532–868	575–922	607–885	723–873
**ActiGraph GT3X+ (Waist)**	6.7 ± 22.7	29.7 ± 33.3	138.2 ± 83.2	328.2 ± 93.9	490.4 ± 86.6	557.5 ± 88.8	604.4 ± 91	646.6 ± 116.9	715.8 ± 140.1	759.2 ± 85.4
Min–Max	0–215	0–170	0–340	0–517	0–622	0–832	0–808	0–872	0–883	477–873
**ActiGraph GT3X+ (Wrist)**	38.9 ± 44.7	118.1 ± 78	227.7 ± 85.7	307.8 ± 106.2	342.7 ± 121.9	363.6 ± 125	368 ± 118	370.7 ± 119.1	378.3 ± 94.1	403.2 ± 56.6
Min–Max	0–227	0–347	0–428	0–532	0–582	0–615	0–593	0–648	0–630	362–603
**ActivPAL**	138.6 ± 119.4	364.6 ± 107.7	447 ± 82.2	497.6 ± 74.6	543.3 ± 70.8	581.5 ± 72.3	620.2 ± 79.8	648.2 ± 109.2	679 ± 178.6	706.7 ± 197.7
Min–Max	0–590	100–597	210–697	257–737	290–703	313–783	337–810	17–823	10–817	30–820
**Digiwalker SW-200**	51.6 ± 62.8	151.8 ± 123.4	312.6 ± 138.8	433.3 ± 105.2	524.1 ± 83.8	577.6 ± 81.8	628.8 ± 87.7	679.5 ± 108	747.8 ± 71.4	774.9 ± 37.9
Min–Max	0–507	0–484	2–635	3–687	106–676	167–811	0–829	6–912	604–879	716–863
**NL-1000**	30.6 ± 52.9	92.3 ± 102.5	297.2 ± 119.5	449.9 ± 86.5	534.6 ± 78.3	575.5 ± 96.8	626.2 ± 71.1	680.2 ± 76.5	743.4 ± 75	771.8 ± 35.8
Min–Max	0–481	0–506	2–651	273–684	0–683	0–828	268–858	571–898	604–879	714–853
**SenseWear**	4.4 ± 6.5	13.5 ± 26.5	60.2 ± 111	131.2 ± 179.5	181 ± 234.1	227.2 ± 265.6	243.3 ± 279.2	282.2 ± 298.7	293 ± 318.6	324.7 ± 320.6
Min–Max	0–58	0–170	0–485	0–567	0–625	10–835	8–692	22–885	0–880	76–822
**StepWatch**	276 ± 101.1	395.1 ± 96.7	448 ± 90.3	496.8 ± 86.9	543.6 ± 88.4	584.8 ± 70.6	612.9 ± 74.7	623.9 ± 87.1	586.1 ± 102.5	580.6 ± 43.8
Min–Max	0–573	0–603	0–663	0–690	0–693	0–690	0–690	0–710	0–723	523–700

Values represent the average number of steps ± standard deviation, unless otherwise indicated.

Supplementary Table 2, Additional File 4. **S**ample sizes (*N*) and number of steps derived from direct observation and each wearable technology by each treadmill speed for children (6–12 years old)

	**Treadmill speed, km/h (mph)**									
	**Slow**				**Normal**				**Fast**	
	**0.8 (0.5)**	**1.6 (1.0)**	**2.4 (1.5)**	**3.2 (2.0)**	**4.0 (2.5)**	**4.8 (3.0)**	**5.6 (3.5)**	**6.4 (4.0)**	**7.2 (4.5)**	**8.0 (5.0)**
N	*53*	*53*	*51*	*49*	*47*	*42*	*34*	*24*	*10*	*0*
**Direct observation**	378.5 ± 83.1	451.3 ± 74.8	503.2 ± 66.9	546.0 ± 58.9	595.6 ± 42.8	630.0 ± 55.5	682.2 ± 59.9	742.5 ± 81.7	809.7 ± 64.2	–
Min–Max	190–603	320–617	407–709	403–736	518–706	508–816	601–850	638–895	659–870	–
**Actical**	39.5 ± 67.6	153.8 ± 91.8	384.4 ± 81.4	533.4 ± 49.9	594.0 ± 39.1	632.7 ± 55.4	684.9 ± 59.9	746.0 ± 90.4	803.2 ± 89.7	–
Min–Max	0–376	0–356	190–548	420–681	523–690	486–840	601–868	626–921	611–885	–
**ActiGraph GT3X+ (Waist)**	13.1 ± 32.3	41.4 ± 35.9	157.0 ± 76.9	345.4 ± 86.2	518.3 ± 65.9	595.5 ± 58.1	654.8 ± 51.7	702.9 ± 101.1	747.5 ± 183.9	–
Min–Max	0–215	0–170	6–308	175–500	338–621	446–831	533–808	528–871	340–883	–
**ActiGraph GT3X+ (Wrist)**	64.5 ± 49.5	146.9 ± 77.6	241.6 ± 79.4	310.3 ± 105.7	350.7 ± 120.6	376.7 ± 120.3	379.7 ± 112.7	379.7 ± 97.4	415.5 ± 30.2	–
Min–Max	3–226	1–346	83–428	36–531	31–581	80–615	96–593	161–586	361–470	–
**ActivPAL**	172.8 ± 132.7	411.8 ± 106.3	496.1 ± 79.8	543.4 ± 71.5	587.1 ± 63.8	621.0 ± 73.3	664.7 ± 81.6	676.7 ± 90.3	721.0 ± 115.6	–
Min–Max	0–590	160–596	283–696	316–736	346–703	366–783	396–810	423–800	420–806	–
**Digiwalker SW-200**	74.1 ± 85.1	192.2 ± 139.7	349.1 ± 148.1	464.0 ± 127.9	551.9 ± 107.9	598.1 ± 121.7	668.1 ± 131.8	719.1 ± 174.3	817.3 ± 58.2	–
Min–Max	0–507	1–484	2–635	3–687	106–676	167–811	0–829	6–912	690–879	–
**NL-1000**	46.4 ± 74.5	128.2 ± 120.8	319.2 ± 103.3	490.1 ± 66.1	583.9 ± 46.5	610.9 ± 112.7	672.4 ± 93.6	741.5 ± 84.4	793.6 ± 85.0	–
Min–Max	0–481	0–506	142–544	314–639	459–683	0–828	268–858	628–898	631–879	–
**SenseWear**	6.3 ± 8.9	20.2 ± 30.9	69.4 ± 108.1	132.6 ± 182.8	195.9 ± 247.5	250.2 ± 284.6	243.2 ± 288.5	298.9 ± 323.0	149.2 ± 260.0	–
Min–Max	0–58	0–141	0–393	0–566	0–625	18–835	23–691	29–885	0–880	–
**StepWatch**	320.7 ± 102.8	446.4 ± 97.9	494.8 ± 92.8	539.3 ± 91.3	582.4 ± 94.2	624.5 ± 31.0	642.0 ± 39.3	623.1 ± 68.6	571.0 ± 46.1	–
Min–Max	0–573	0–603	0–663	0–690	0–693	556–690	543–690	426–696	483–653	–

Values represent the average number of steps ± standard deviation, unless otherwise indicated.

Supplementary Table 3, Additional File 4. **S**ample sizes (*N*) and number of steps derived from direct observation and each wearable technology by each treadmill speed for adolescents (13–17 years old)

	Treadmill speed, km/h (mph)									
	Slow				Normal				Fast	
	0.8 (0.5)	1.6 (1.0)	2.4 (1.5)	3.2 (2.0)	4.0 (2.5)	4.8 (3.0)	5.6 (3.5)	6.4 (4.0)	7.2 (4.5)	8.0 (5.0)
N	40	40	40	40	39	39	36	34	25	6
**Direct observation**	290.1 ± 68.9	361.1 ± 58.8	418.8 ± 42	474.1 ± 33.1	525.5 ± 32.6	568.8 ± 31.6	607.4 ± 32.8	662.3 ± 52.7	746 ± 66.6	750.8 ± 32
Min–Max	162–453	255–486	338–498	396–536	474–621	512–640	535–666	587–822	626–843	712–802
**Actical**	4.8 ± 13	124.8 ± 92.9	314 ± 85.6	456.1 ± 53.2	518.2 ± 41.1	566.3 ± 31.7	609.9 ± 33.1	664.6 ± 56.8	751.8 ± 71	757.5 ± 31
Min–Max	0–55	0–318	142–555	230–538	343–567	482–612	532–672	575–833	615–860	723–805
**ActiGraph GT3X+ (Waist)**	1.2 ± 2.7	21.1 ± 29.1	124.6 ± 80.9	312.5 ± 91.9	477.8 ± 70.6	539.4 ± 69.4	586.5 ± 51.2	629.2 ± 74.8	741.7 ± 68.3	711.9 ± 119.5
Min–Max	0–13	0–127	0–305	77–503	267–563	292–600	408–655	463–833	610–842	477–807
**ActiGraph GT3X+ (Wrist)**	15.6 ± 17.4	92.7 ± 68.5	214.2 ± 92.1	302.4 ± 112.8	334.3 ± 127.8	359.1 ± 133.5	355.1 ± 116.3	346.6 ± 120.2	363.3 ± 79.7	419.2 ± 90.8
Min–Max	0–68	5–292	2–403	2–478	5–553	2–580	3–568	7–618	87–478	367–603
**ActivPAL**	120.1 ± 102.4	335.2 ± 98.7	407.8 ± 65.4	460.6 ± 65.5	507.4 ± 68.4	550.2 ± 71.1	591.1 ± 83.6	621.7 ± 140.9	636.3 ± 241.4	708.9 ± 120.8
Min–Max	0–443	100–500	210–500	257–540	290–567	313–613	337–747	17–823	10–817	470–803
**Digiwalker SW-200**	32.9 ± 20	127.7 ± 98.7	284.6 ± 116.2	412.5 ± 72.7	506.3 ± 46	566.8 ± 29.7	610.1 ± 30.1	665.4 ± 56	747.6 ± 65	756.5 ± 30.4
Min–Max	8–95	3–426	90–565	247–626	369–587	504–636	558–666	600–825	626–846	716–797
**NL-1000**	17.9 ± 10.3	67.4 ± 77.9	270 ± 124.3	418.1 ± 91.1	511.1 ± 43.8	562.6 ± 29.1	601.9 ± 36	657.4 ± 54.5	749 ± 71.1	754.8 ± 30.6
Min–Max	8–60	0–292	13–521	274–684	380–638	512–611	516–663	574–829	614–864	714–799
**SenseWear**	3.3 ± 2.3	9.8 ± 26.3	47.1 ± 103.9	106.5 ± 160.1	139.9 ± 209.3	190.4 ± 245.9	226.9 ± 272.9	256.3 ± 278.5	311.8 ± 319.1	369.6 ± 318.7
Min–Max	0–8	0–170	0–485	0–478	0–547	10–587	8–617	22–715	32–813	97–780
**StepWatch**	248.9 ± 83.5	364.1 ± 57.6	419.9 ± 41.8	473.7 ± 33.8	524.7 ± 29	569.3 ± 28.9	609.7 ± 33.4	645.3 ± 36.5	596.1 ± 61.6	596.7 ± 52.5
Min–Max	97–450	273–493	357–507	400–543	477–570	517–613	533–677	560–710	517–723	557–700

Values represent the average number of steps ± standard deviation, unless otherwise indicated.

Supplementary Table 4, Additional File 4. **S**ample sizes (*N*) and number of steps derived from direct observation and each wearable technology by each treadmill speed for young adults (18–20 years old)

	**Treadmill speed, km/h (mph)**									
	**Slow**				**Normal**				**Fast**	
	**0.8 (0.5)**	**1.6 (1.0)**	**2.4 (1.5)**	**3.2 (2.0)**	**4.0 (2.5)**	**4.8 (3.0)**	**5.6 (3.5)**	**6.4 (4.0)**	**7.2 (4.5)**	**8.0 (5.0)**
N	24	24	24	24	24	23	23	23	18	10
**Direct observation**	248.6 ± 56.9	335.4 ± 58.1	408.5 ± 52.9	466 ± 33.3	516.9 ± 28.1	562.4 ± 24.8	604.5 ± 25	657.8 ± 52.5	710.9 ± 55.6	768.8 ± 44.5
Min–Max	161–427	273–532	347–575	414–539	472–570	515–599	547–637	577–771	611–798	693–858
**Actical**	4.2 ± 13.8	168.8 ± 104.4	338.3 ± 86.5	459.7 ± 36.4	518.7 ± 28.4	563.3 ± 25.2	600.5 ± 26.1	658.9 ± 55	712 ± 57.7	788.2 ± 42.7
Min–Max	0–65	0–367	123–478	388–533	470–572	512–607	543–645	578–778	607–800	732–873
**ActiGraph GT3X+ (Waist)**	1.7 ± 6.8	18.2 ± 25.7	120.9 ± 94.4	319 ± 109.3	456.5 ± 124.6	518.8 ± 132	557.9 ± 140.4	613.7 ± 159.8	662.2 ± 176	787.5 ± 42.9
Min–Max	0–33	0–113	0–340	0–517	0–572	0–607	0–643	0–780	0–800	732–873
**ActiGraph GT3X+ (Wrist)**	21.1 ± 34.9	96.8 ± 74.7	220.8 ± 86.6	311.6 ± 99.7	340.4 ± 118.7	347.3 ± 121.6	371 ± 131.2	397 ± 135.1	378.4 ± 128.4	393.7 ± 21.9
Min–Max	0–160	0–265	0–352	0–467	0–493	0–530	0–558	0–648	0–630	362–437
**ActivPAL**	94 ± 94.6	309.6 ± 81	407.8 ± 52.3	465.8 ± 35.3	516 ± 31.8	562.6 ± 25	599.9 ± 25.2	657.8 ± 54.8	715 ± 55.3	705.3 ± 238.8
Min–Max	0–377	153–540	347–580	407–543	457–573	513–600	547–640	577–777	607–793	30–820
**Digiwalker SW-200**	33 ± 26.2	102.8 ± 94.2	281.5 ± 139.9	405.2 ± 85.1	498.4 ± 60.4	558.3 ± 27.9	600.1 ± 29.4	659 ± 58.9	709.5 ± 58.7	786 ± 38.9
Min–Max	11–108	0–287	16–541	166–581	342–592	507–599	539–642	575–784	604–801	727–863
**NL-1000**	16.7 ± 17.6	54.4 ± 64.7	295.8 ± 138	421 ± 84.8	476.3 ± 110.2	532.9 ± 119	595.8 ± 26.7	650.1 ± 59.4	707.8 ± 58.2	782 ± 36.1
Min–Max	0–96	0–255	2–651	273–581	0–566	0–600	540–641	571–777	604–797	732–853
**SenseWear**	2 ± 1.9	4.7 ± 4	62.7 ± 130.1	169.4 ± 202.9	218.6 ± 245.2	247.8 ± 266.7	269 ± 285.4	302.9 ± 311.7	346.7 ± 339.5	297.8 ± 335.8
Min–Max	0–6	0–17	0–478	9–528	12–545	16–592	20–620	25–780	28–812	76–822
**StepWatch**	222.2 ± 83.4	333.8 ± 90.3	395.3 ± 98.2	448.5 ± 101.2	498.2 ± 109.9	538.7 ± 120.2	575.1 ± 128.1	593.2 ± 137.6	580.6 ± 159.1	571 ± 37.3
Min–Max	0–433	0–543	0–587	0–547	0–570	0–603	0–640	0–677	0–700	523–633

Values represent the average number of steps ± standard deviation, unless otherwise indicated.
