# Supplementary material for: A catalog of validity indices for step counting wearable technologies during treadmill walking: the CADENCE-Kids study
Source: Int J Behav Nutr Phys Act. 2021 Jul 16;18:97. doi: 10.1186/s12966-021-01167-y (PMC8283935; doi:10.1186/s12966-021-01167-y)
Supplement: Supplementary file 6 — Additional file 6. Figures for descriptive statistics representing accuracy, precision and bias for each wearable technology. [file 12966_2021_1167_MOESM6_ESM.pdf]

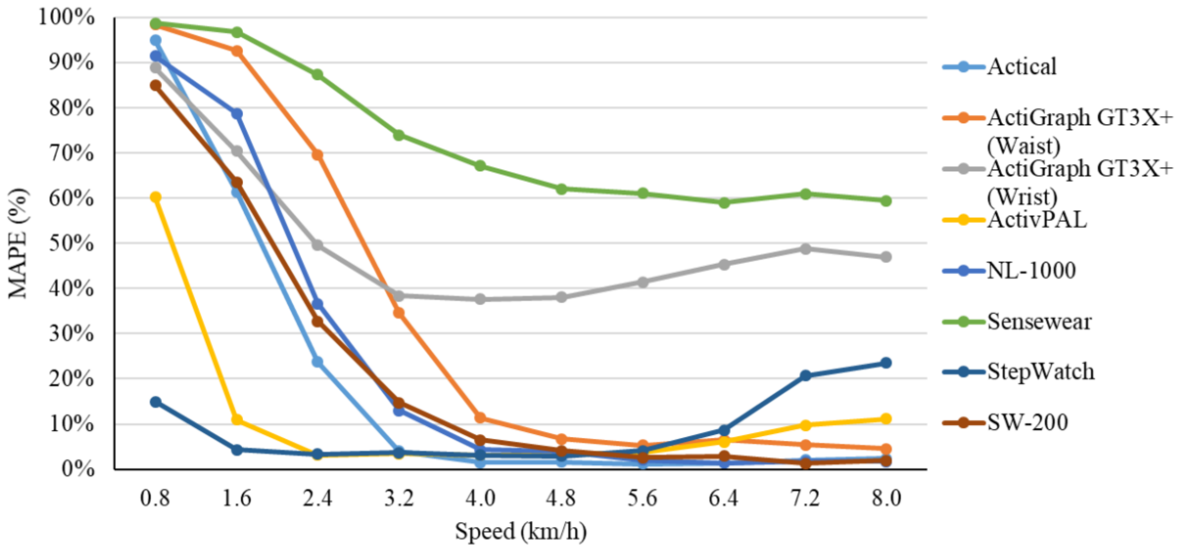

Supplementary Figure 1, Additional file 6. Mean absolute percentage error (MAPE) of each wearable technology across walking speeds. Each line is representative of grouped average for a single technology. Decreases in MAPE indicate improved accuracy.

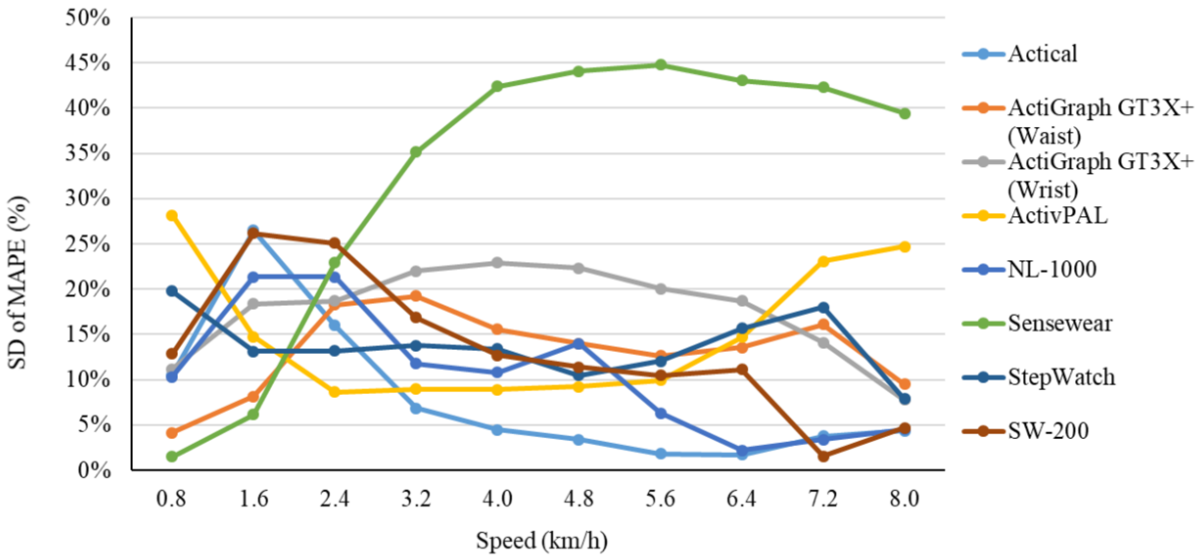

Supplementary Figure 2, Additional file 6. Standard deviation (SD) of mean absolute percentage error (MAPE) of each wearable technology across walking speeds. Each line is representative of grouped average for a single technology. Decrease in SD indicates more precise measures.

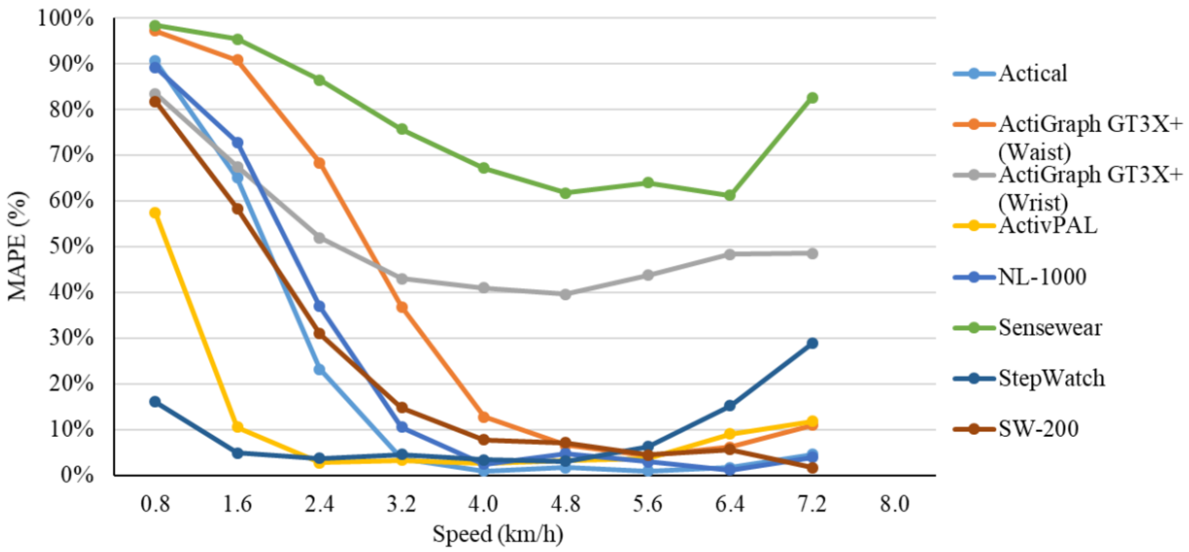

Supplementary Figure 3, Additional file 6. Mean absolute percentage error (MAPE) of each wearable technology across walking speeds for children (6–12 years of age). Each line is representative of grouped average for a single technology. Decreases in MAPE indicate improved accuracy.

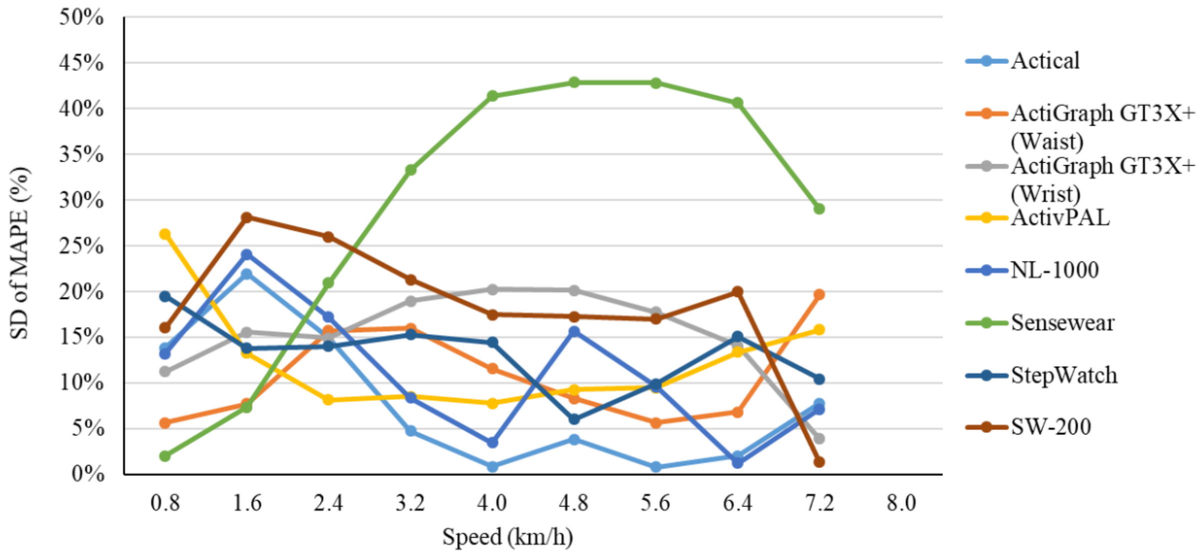

Supplementary Figure 4, Additional file 6. Standard deviation (SD) of mean absolute percentage error (MAPE) of each wearable technology across walking speeds for children (6–12 years of age). Each line is representative of grouped average for a single technology. Decrease in SD indicates more precise measures.

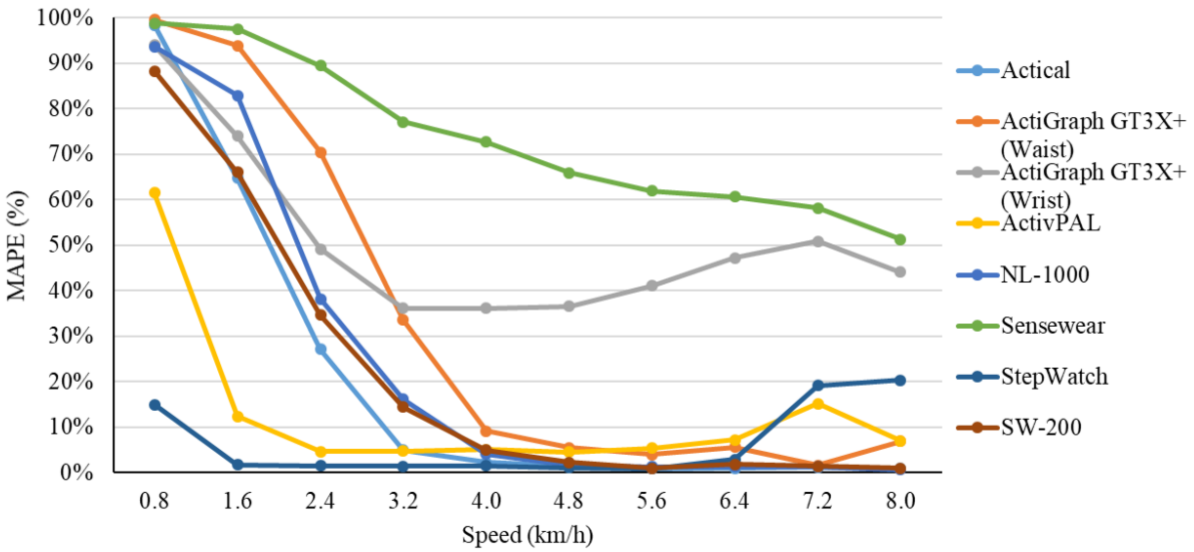

Supplementary Figure 5, Additional file 6. Mean absolute percentage error (MAPE) of each wearable technology across walking speeds for adolescents (13–17 years of age). Each line is representative of grouped average for a single technology. Decreases in MAPE indicate improved accuracy.

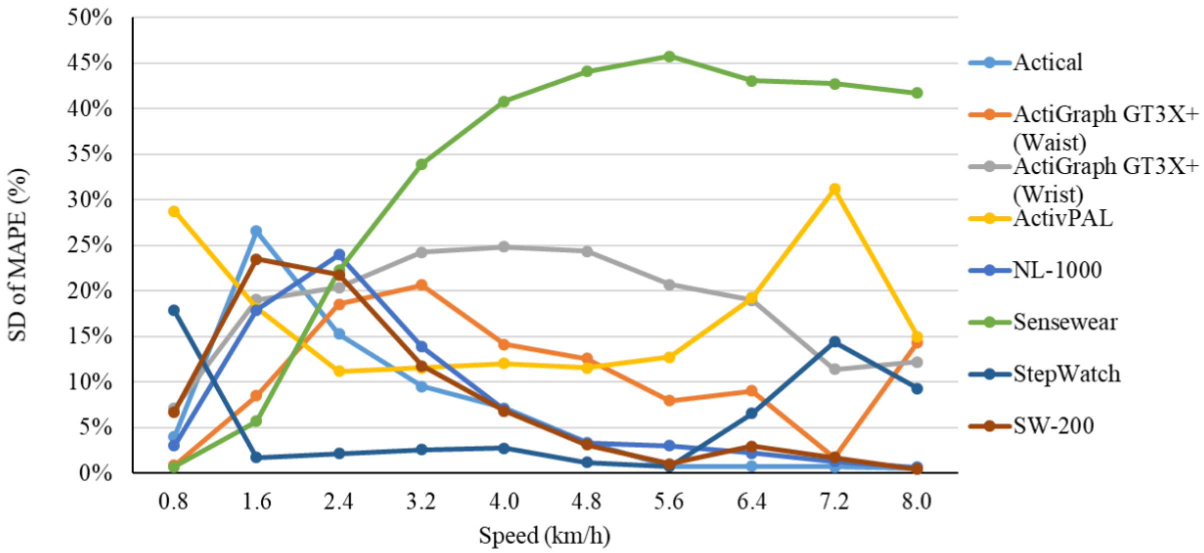

Supplementary Figure 6, Additional file 6. Standard deviation (SD) of mean absolute percentage error (MAPE) of each wearable technology across walking speeds for adolescents (13–17 years of age). Each line is representative of grouped average for a single technology. Decrease in SD indicates more precise measures.

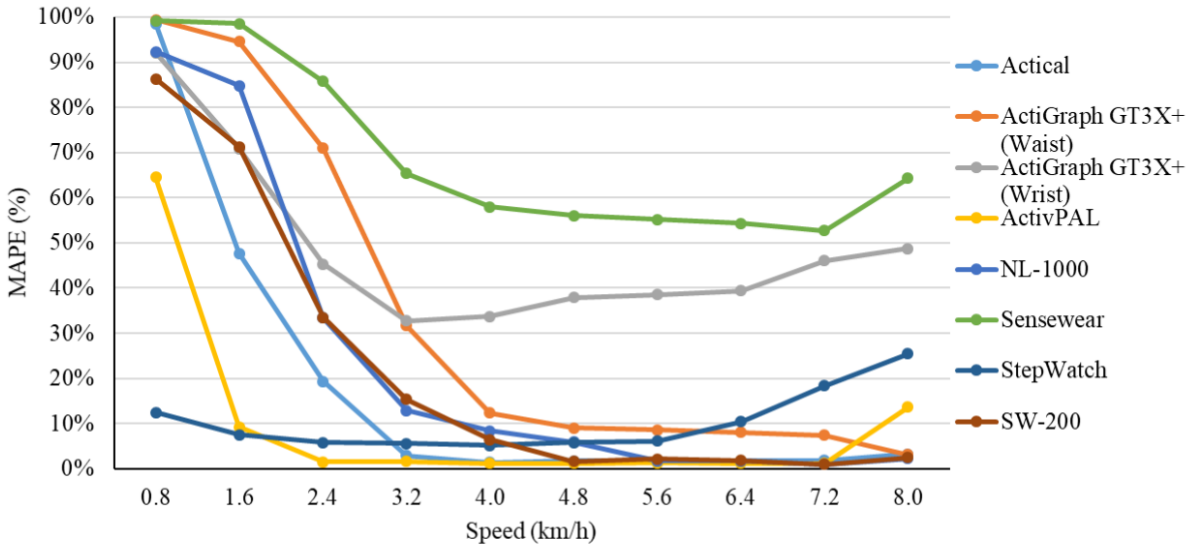

Supplementary Figure 7, Additional file 6. Mean absolute percentage error (MAPE) of each wearable technology across walking speeds for young adults (18–20 years of age). Each line is representative of grouped average for a single technology. Decreases in MAPE indicate improved accuracy.

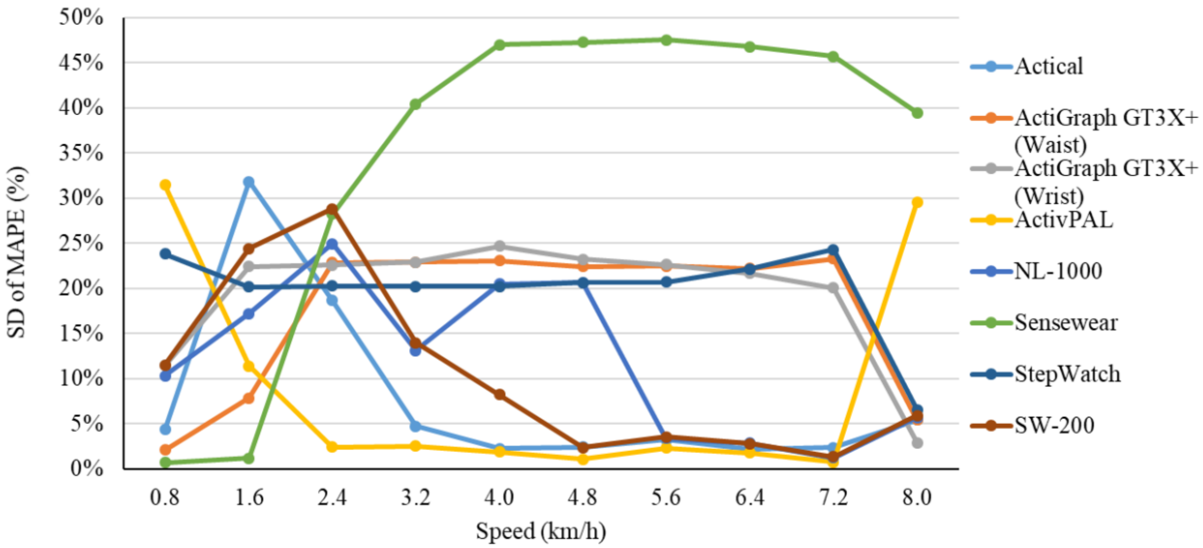

Supplementary Figure 8, Additional file 6. Standard deviation (SD) of mean absolute percentage error (MAPE) of each wearable technology across walking speeds for young adults (18–20 years of age). Each line is representative of grouped average for a single technology. Decrease in SD indicates more precise measures.

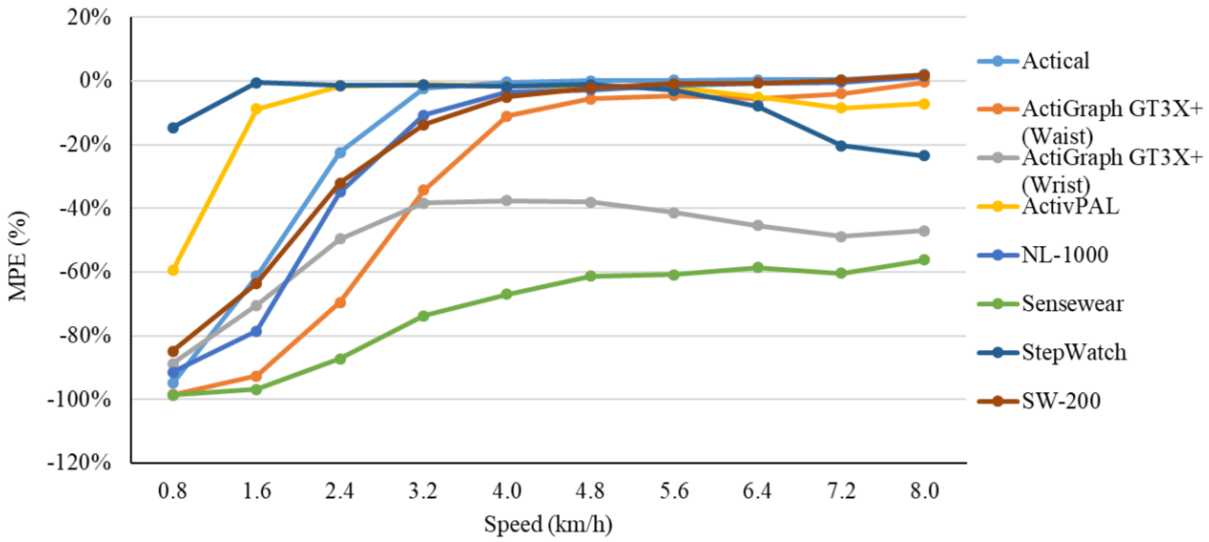

Supplementary Figure 9, Additional file 6. Mean percentage error (MPE) of each wearable technology across walking speeds. Each line is representative of grouped average for a single technology. MPE values closer to 0% indicate improved bias.

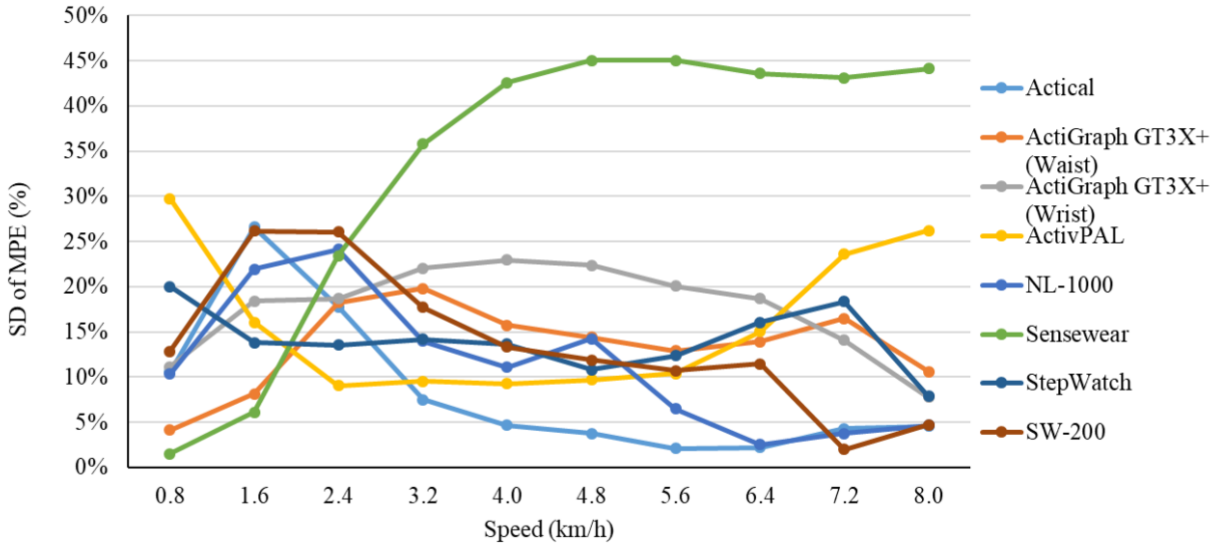

Supplementary Figure 10, Additional file 6. Standard deviation (SD) of mean percentage error (MPE) of each wearable technology across walking speeds. Each line is representative of grouped average for a single technology. Decrease in SD indicates more precise measures.

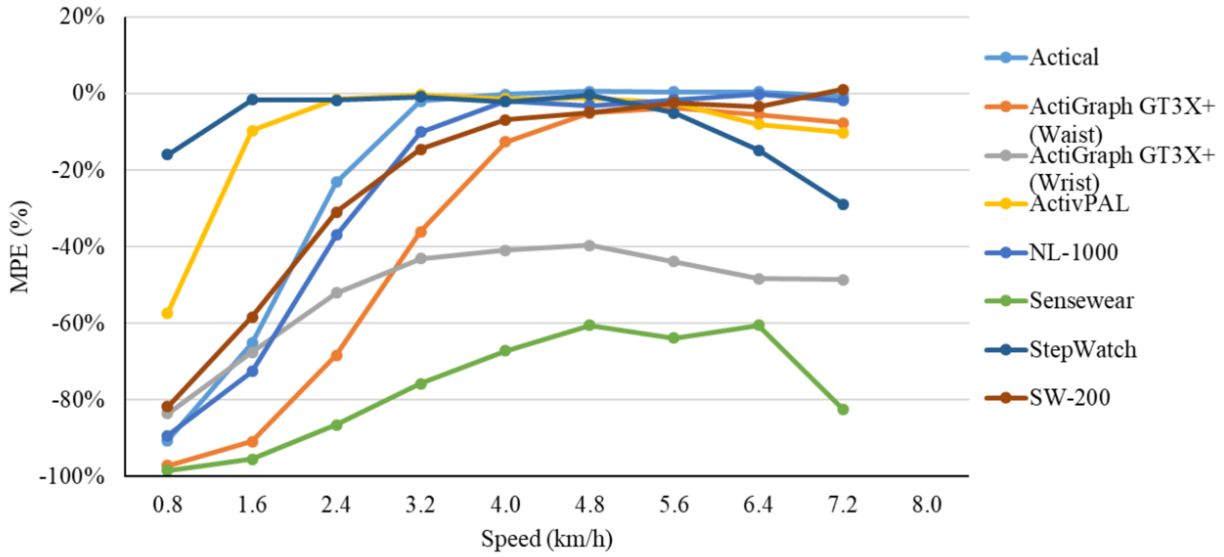

Supplementary Figure 11, Additional file 6. Mean percentage error (MPE) of each wearable technology across walking speeds for children (6–12 years of age). Each line is representative of grouped average for a single technology. MPE values closer to 0% indicate improved bias.

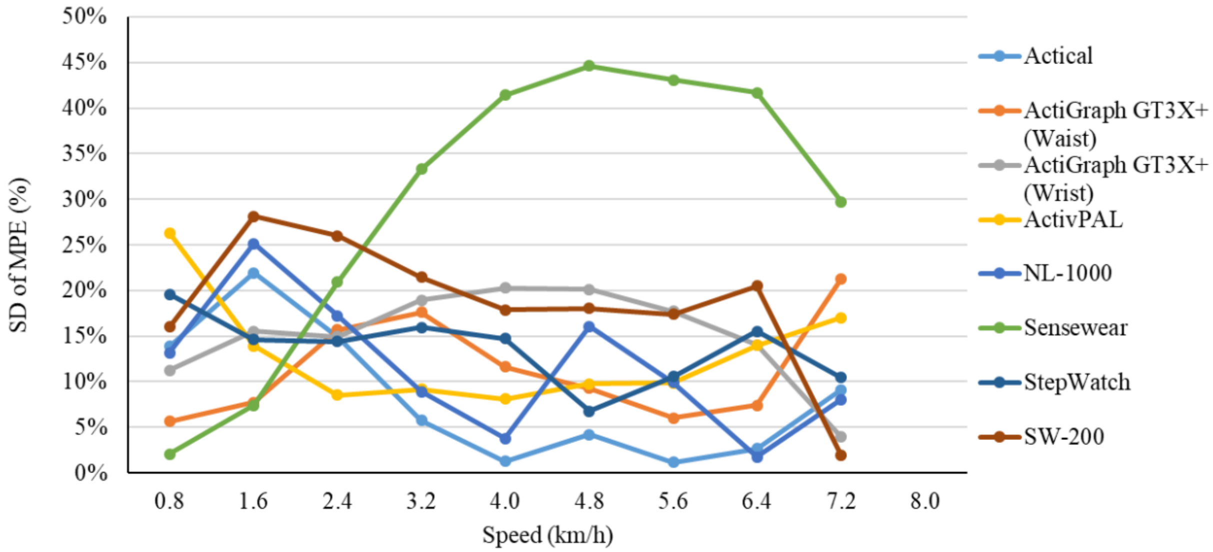

Supplementary Figure 12, Additional file 6. Standard deviation (SD) of mean percentage error (MPE) of each wearable technology across walking speeds for children (6–12 years of age). Each line is representative of grouped average for a single technology. Decrease in SD indicates more precise measures.

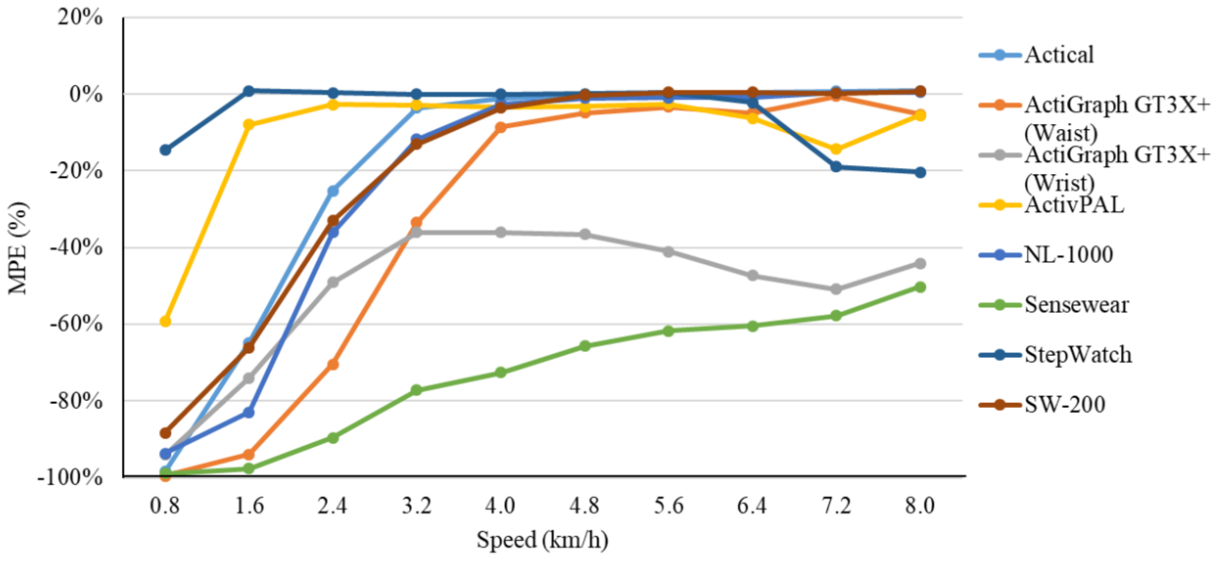

Supplementary Figure 13, Additional file 6. Mean percentage error (MPE) of each wearable technology across walking speeds for adolescents (13–17 years of age). Each line is representative of grouped average for a single technology. MPE values closer to 0% indicate improved bias.

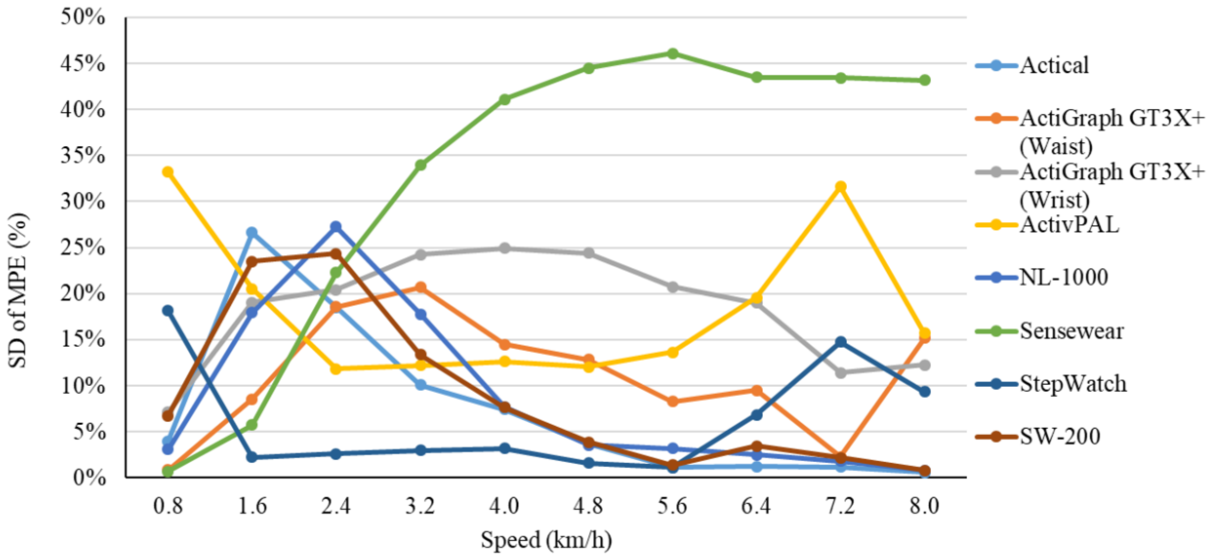

Supplementary Figure 14, Additional file 6. Standard deviation (SD) of mean percentage error (MPE) of each wearable technology across walking speeds for adolescents (13–17 years of age). Each line is representative of grouped average for a single technology. Decrease in SD indicates more precise measures.

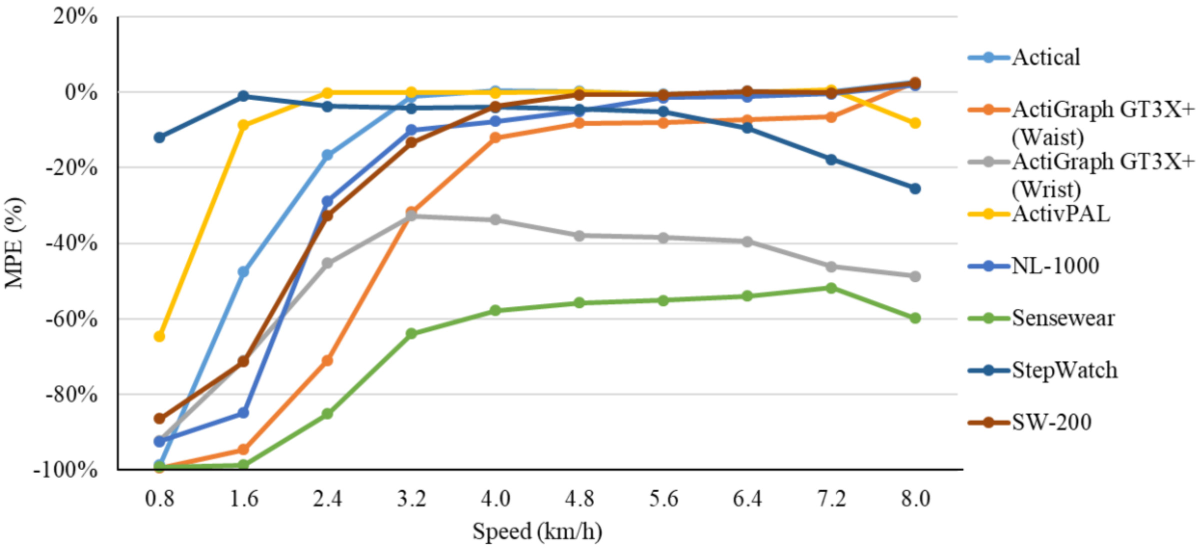

Supplementary Figure 15, Additional file 6. Mean percentage error (MPE) of each wearable technology across walking speeds for young adults (18–20 years of age). Each line is representative of grouped average for a single technology. MPE values closer to 0% indicate improved bias.

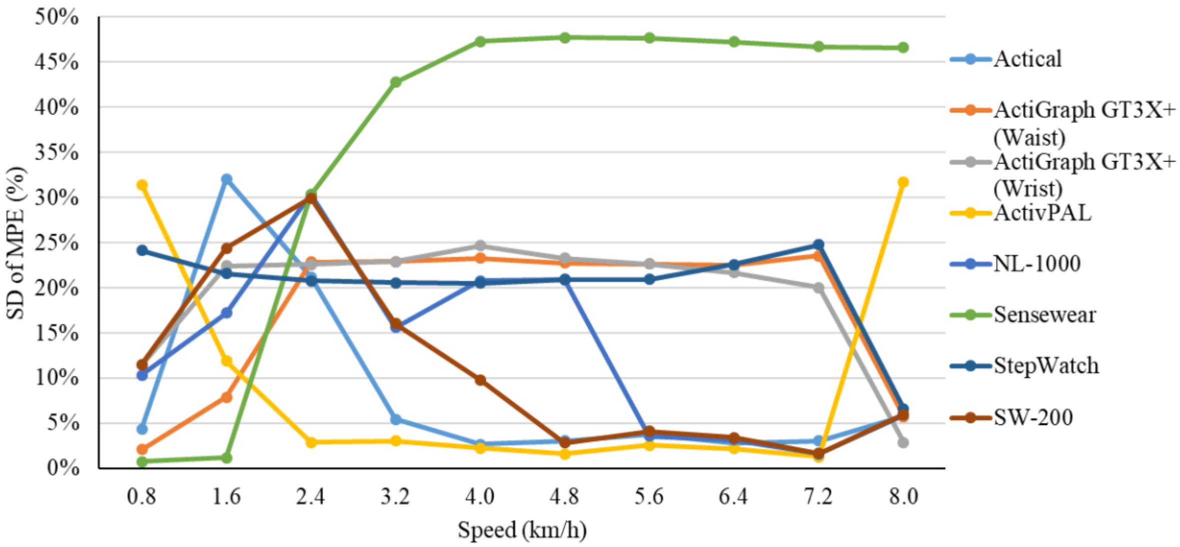

Supplementary Figure 16, Additional file 6. Standard deviation (SD) of mean percentage error (MPE) of each wearable technology across walking speeds for young adults (18–20 years of age). Each line is representative of grouped average for a single technology. Decrease in SD indicates more precise measures.
