# Supplementary material for: A catalog of validity indices for step counting wearable technologies during treadmill walking: the CADENCE-Kids study
Source: Int J Behav Nutr Phys Act. 2021 Jul 16;18:97. doi: 10.1186/s12966-021-01167-y (PMC8283935; doi:10.1186/s12966-021-01167-y)
Supplement: Supplementary file 7 — Additional file 7 Tabular and graphical representations of correlation coefficients (r) of the relationship between directly observed steps and steps derived from wearable technologies. [file 12966_2021_1167_MOESM7_ESM.pdf]

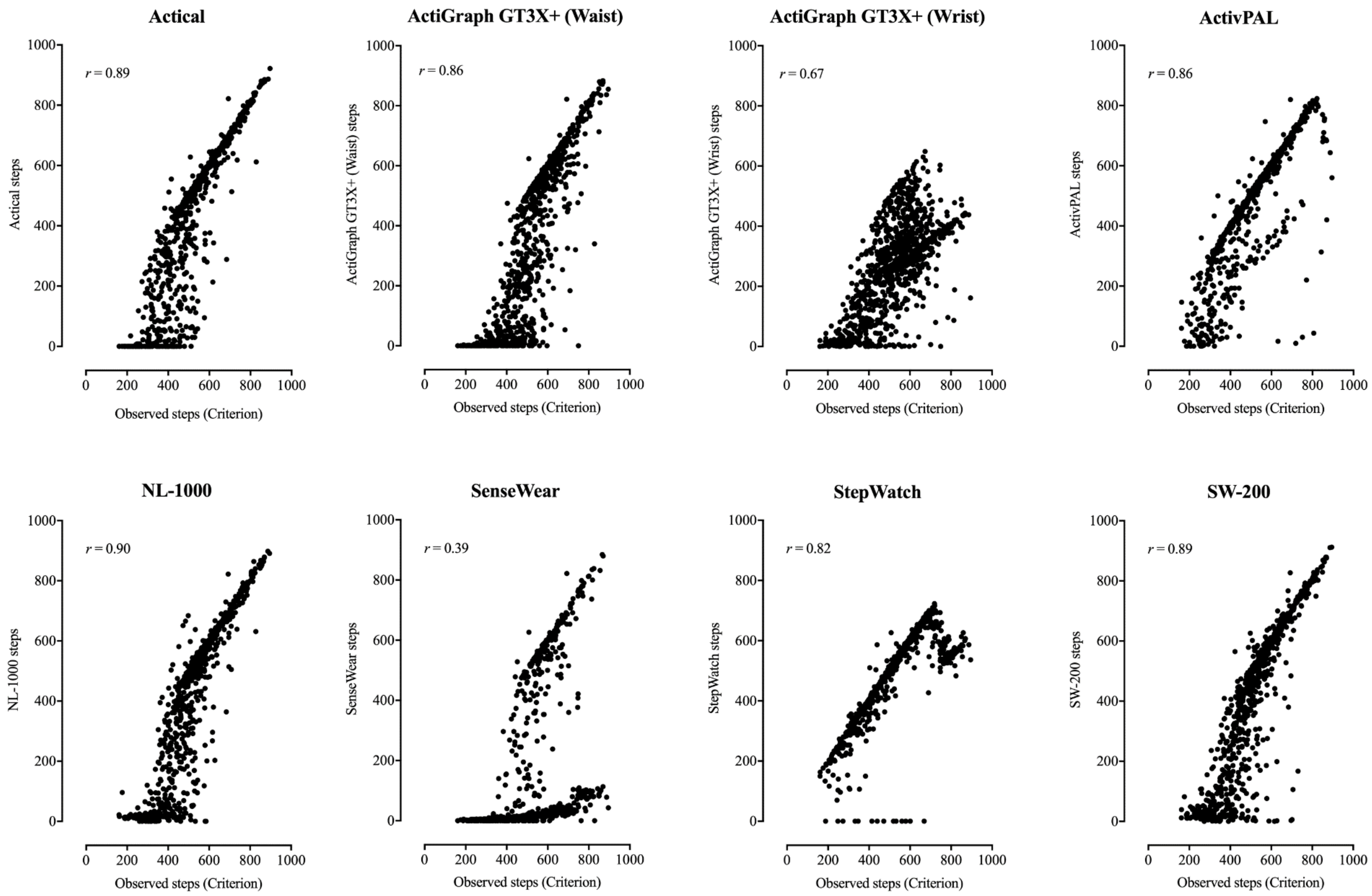
Actical
r = 0.89
Actical steps
Observed steps (Criterion)
ActiGraph GT3X+ (Waist)
r = 0.86
ActiGraph GT3X+ (Waist) steps
Observed steps (Criterion)
ActiGraph GT3X+ (Wrist)
r = 0.67
ActiGraph GT3X+ (Wrist) steps
Observed steps (Criterion)
ActivPAL
r = 0.86
ActivPAL steps
Observed steps (Criterion)
NL-1000
r = 0.90
NL-1000 steps
Observed steps (Criterion)
SenseWear
r = 0.39
SenseWear steps
Observed steps (Criterion)
StepWatch
r = 0.82
StepWatch steps
Observed steps (Criterion)
SW-200
r = 0.89
SW-200 steps
Observed steps (Criterion)
0
200
400
600
800
1000

**Supplementary Figure 1, Additional File 7**. Representation of the correlation between directly observed steps and steps derived from wearable technologies across all walking bouts. The X-Y scatterplot is representative of how tightly the wearable technology step counts hold to a linear relationship to directly observed steps across all walking bouts. X axis represents directly observed steps, and Y axis represents steps derived from wearable technology. Correlation coefficients (*r*) closer to 1.0 indicate tighter relationship (more precise) to directly observed steps.
