# Supplementary material for: A catalog of validity indices for step counting wearable technologies during treadmill walking: the CADENCE-Kids study
Source: Int J Behav Nutr Phys Act. 2021 Jul 16;18:97. doi: 10.1186/s12966-021-01167-y (PMC8283935; doi:10.1186/s12966-021-01167-y)
Supplement: Supplementary file 8 — Additional file 8. Figures of the effect of speed, wear location, and age on the overall precision of wearable technologies step counting ability. [file 12966_2021_1167_MOESM8_ESM.pdf]

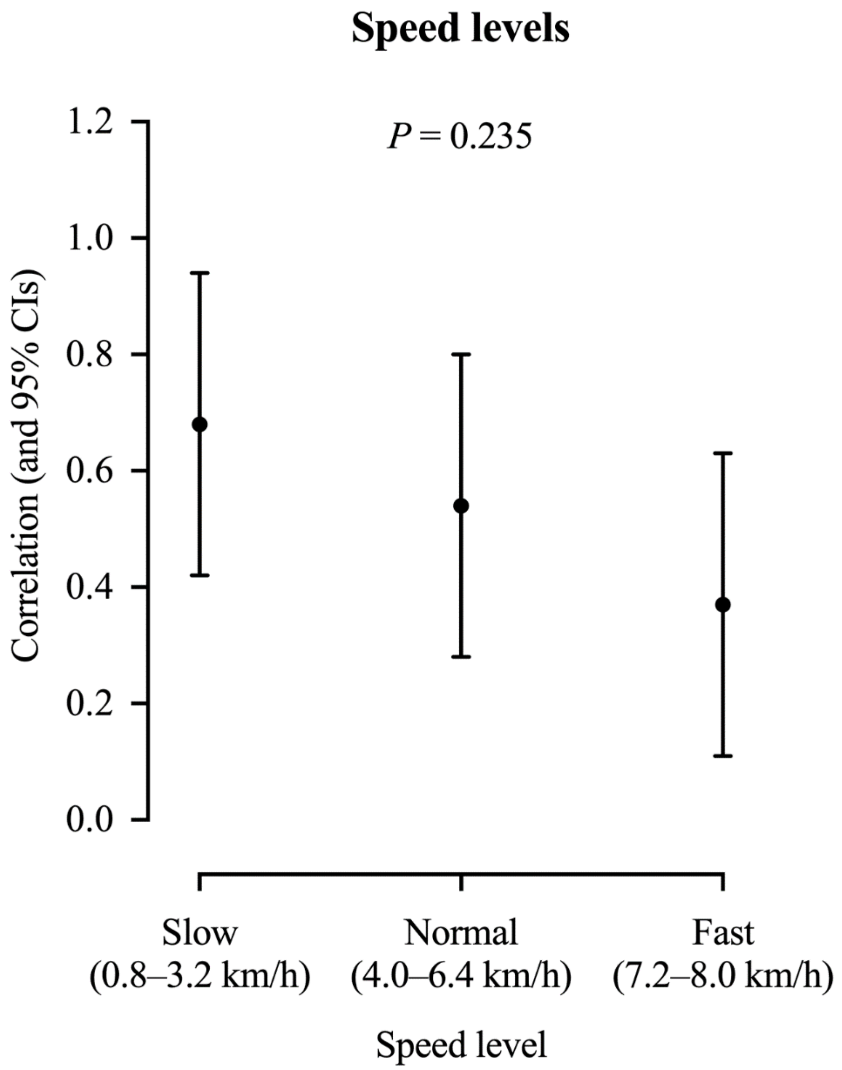

Supplementary Figure 1, Additional File 8. Effect of speed on overall precision of wearable technologies' step counting ability. Each point represents correlation coefficients averaged across wearable technologies and each speed range (i.e., slow, normal or fast), with corresponding 95% confidence intervals (CIs) extending above and below that point estimate. Correlation coefficients closer to 1.0 indicate tighter relationship (more precise) to directly-observed steps, i.e., increased precision. 95% CIs that do not overlap indicate significant differences, while those that do overlap indicate no significant differences.

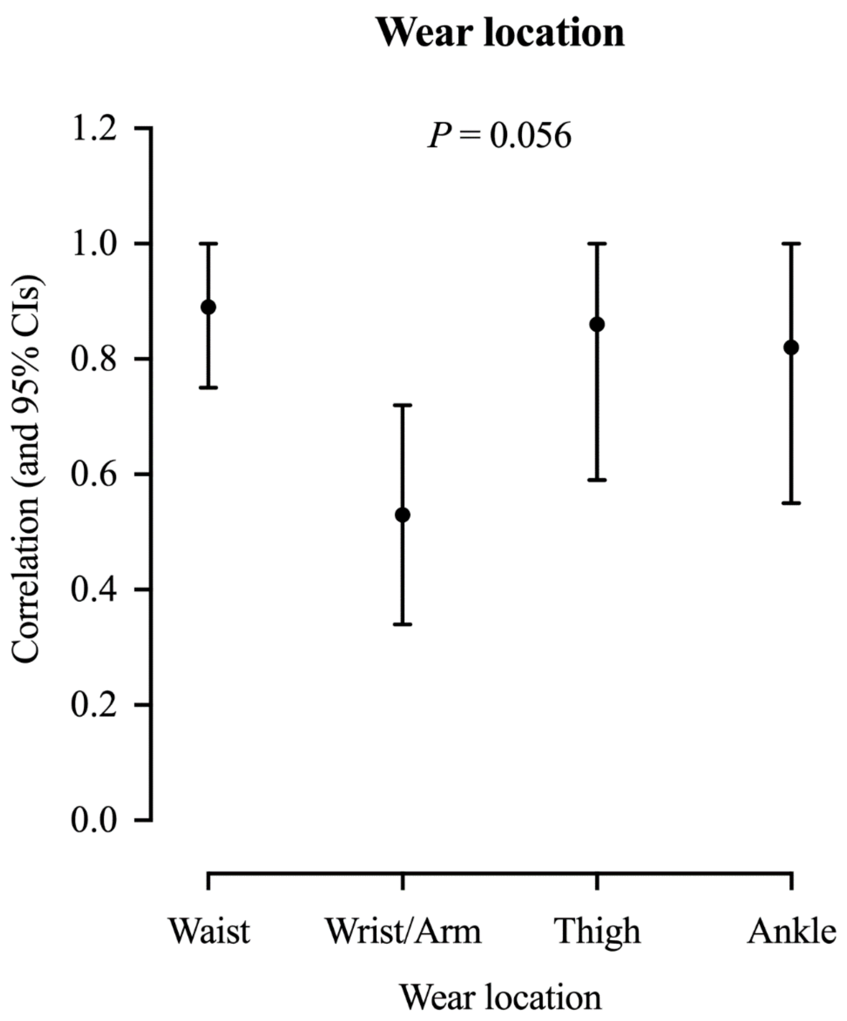

Supplementary Figure 2, Additional File 8. Effect of wear location on overall precision of wearable technologies' step counting ability. Each point represents correlation coefficients averaged across technologies respective to each wear location for all walking bouts, with corresponding 95% confidence intervals (CIs) extending above and below that point estimate. Correlation coefficients closer to 1.0 indicate greater precision. Where 95% CIs do not overlap, there are significant differences between locations.

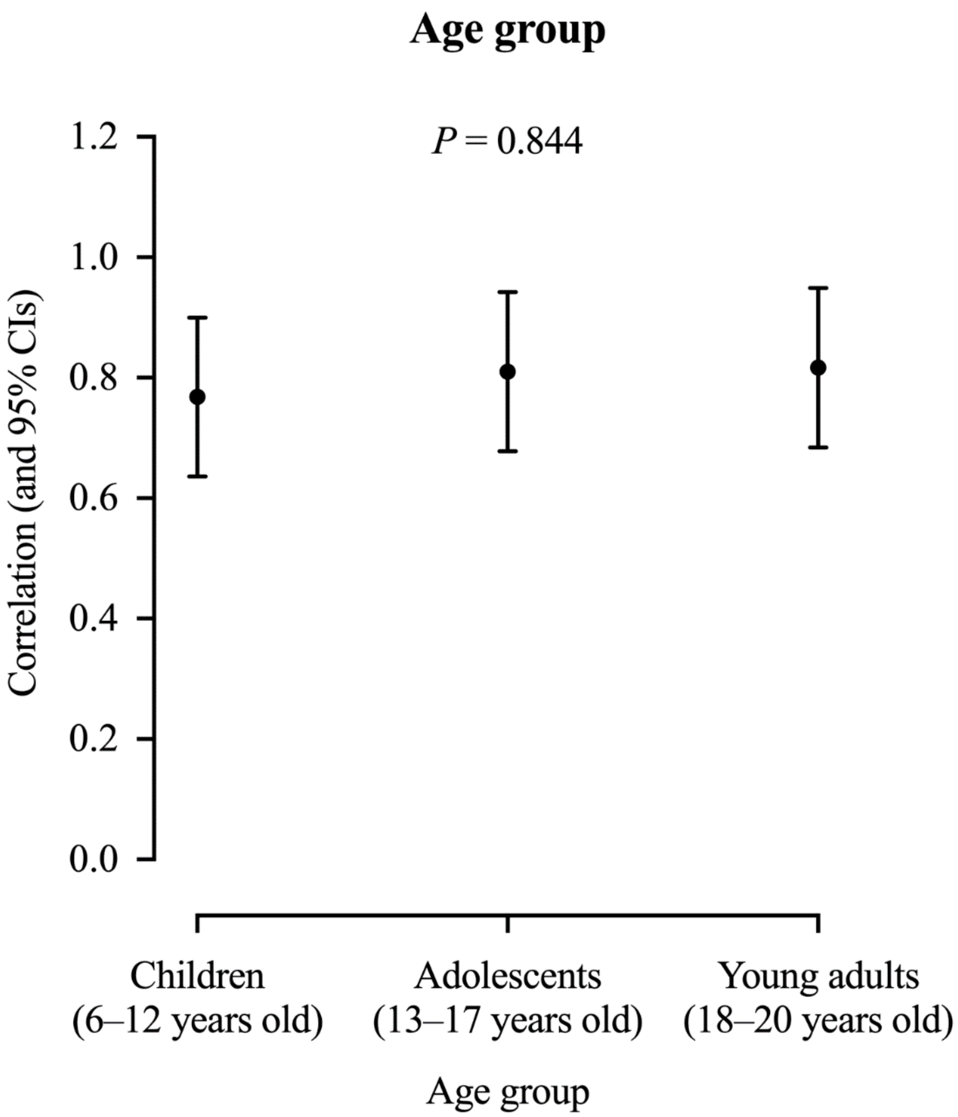

Supplementary Figure 3, Additional File 8. Effect of age on overall precision of wearable technologies' step counting ability. Each point represents correlation coefficients averaged across technologies respective to each age group for all walking bouts, with corresponding 95% confidence intervals (CIs) extending above and below that point estimate. Correlation coefficients closer to 1.0 indicate greater precision. Where 95% CIs do not overlap, there are significant differences between locations.
