# Supplementary material for: A catalog of validity indices for step counting wearable technologies during treadmill walking: the CADENCE-Kids study
Source: Int J Behav Nutr Phys Act. 2021 Jul 16;18:97. doi: 10.1186/s12966-021-01167-y (PMC8283935; doi:10.1186/s12966-021-01167-y)
Supplement: Supplementary file 9 — Additional file 9. Figures of the effect of speed, wear location, and age on bias of wearable technologies step counting ability. [file 12966_2021_1167_MOESM9_ESM.pdf]

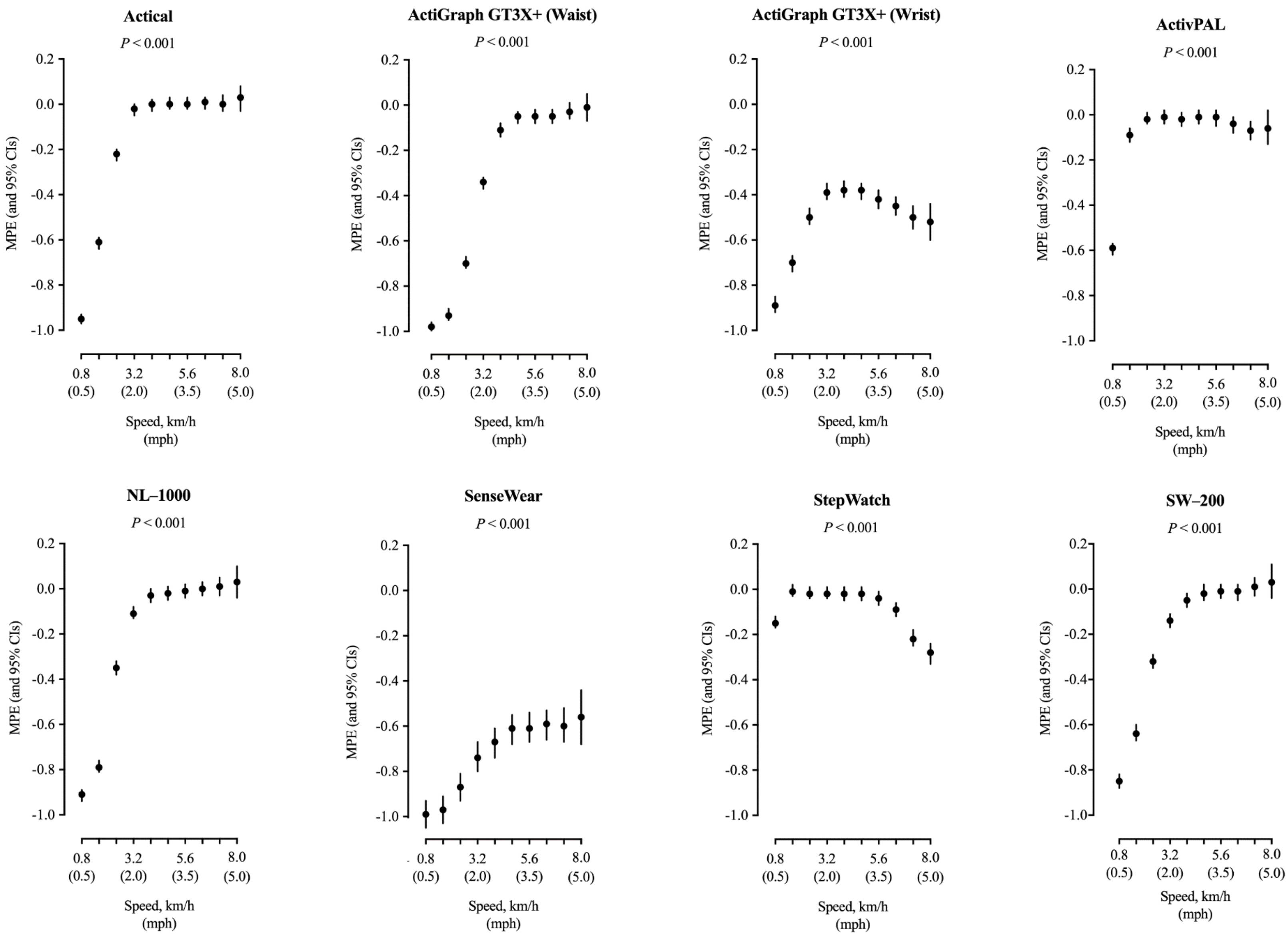
Actical
P < 0.001
ActiGraph GT3X+ (Waist)
P < 0.001
ActiGraph GT3X+ (Wrist)
P < 0.001
ActivPAL
P < 0.001
NL–1000
P < 0.001
SenseWear
P < 0.001
StepWatch
P < 0.001
SW–200
P < 0.001
MPE (and 95% CIs)
0.2
0.0
-0.2
-0.4
-0.6
-0.8
-1.0
0.8 (0.5)
3.2 (2.0)
5.6 (3.5)
8.0 (5.0)
Speed, km/h (mph)

Supplementary Figure 1, Additional File 9. Effect of speed on bias (mean percentage error, MPE) of wearable technology's step counting ability. MPE (%) and corresponding 95% confidence intervals (CIs) respective to each technology are plotted across speeds. Each point represents grouped averages of MPE values, with 95% CIs estimated using mixed effect models and extending above and below that point estimate. MPE values closer to 0% are indicative of improved bias and negative values mean undercounting. 95% CIs that do not overlap indicate significant differences, while those that do overlap indicate no significant differences. Overall likelihood ratio test *P* value is reported for the effect of all speeds on MPE.

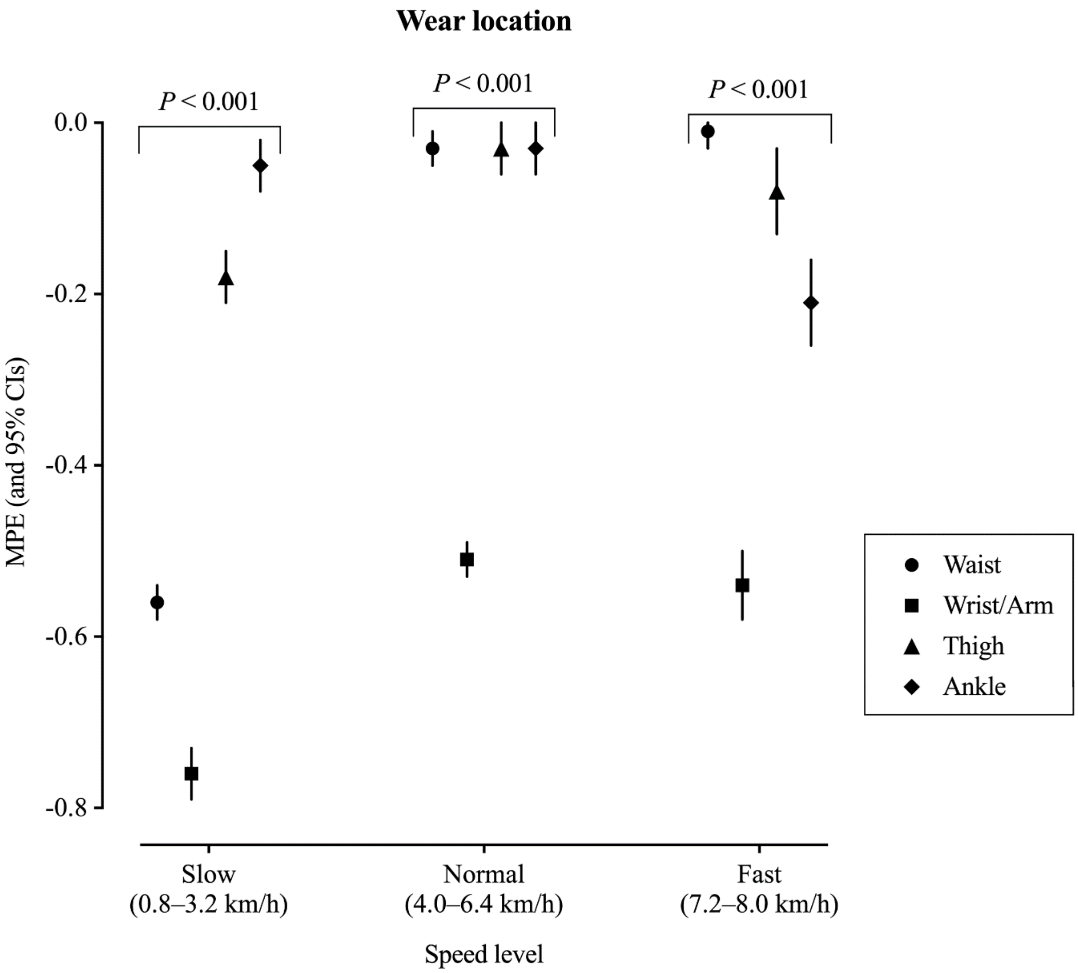

Supplementary Figure 2, Additional File 9. Effect of wear location on bias (mean percentage error, MPE) of wearable technologies' step counting ability. MPE (%) and corresponding 95% confidence intervals (CIs; estimated using mixed effect models) of each wear location are presented at slow, normal, and fast walking speeds. MPE values were averaged across devices respective to each wear location for slow, normal, and fast walking speeds. MPE values closer to 0% are indicative of improved bias and negative values mean undercounting. 95% CIs that do not overlap indicate significant differences, while those that do overlap indicate no significant differences. *P* value is reported for the effect of wear location on MPE for each specific speed level.

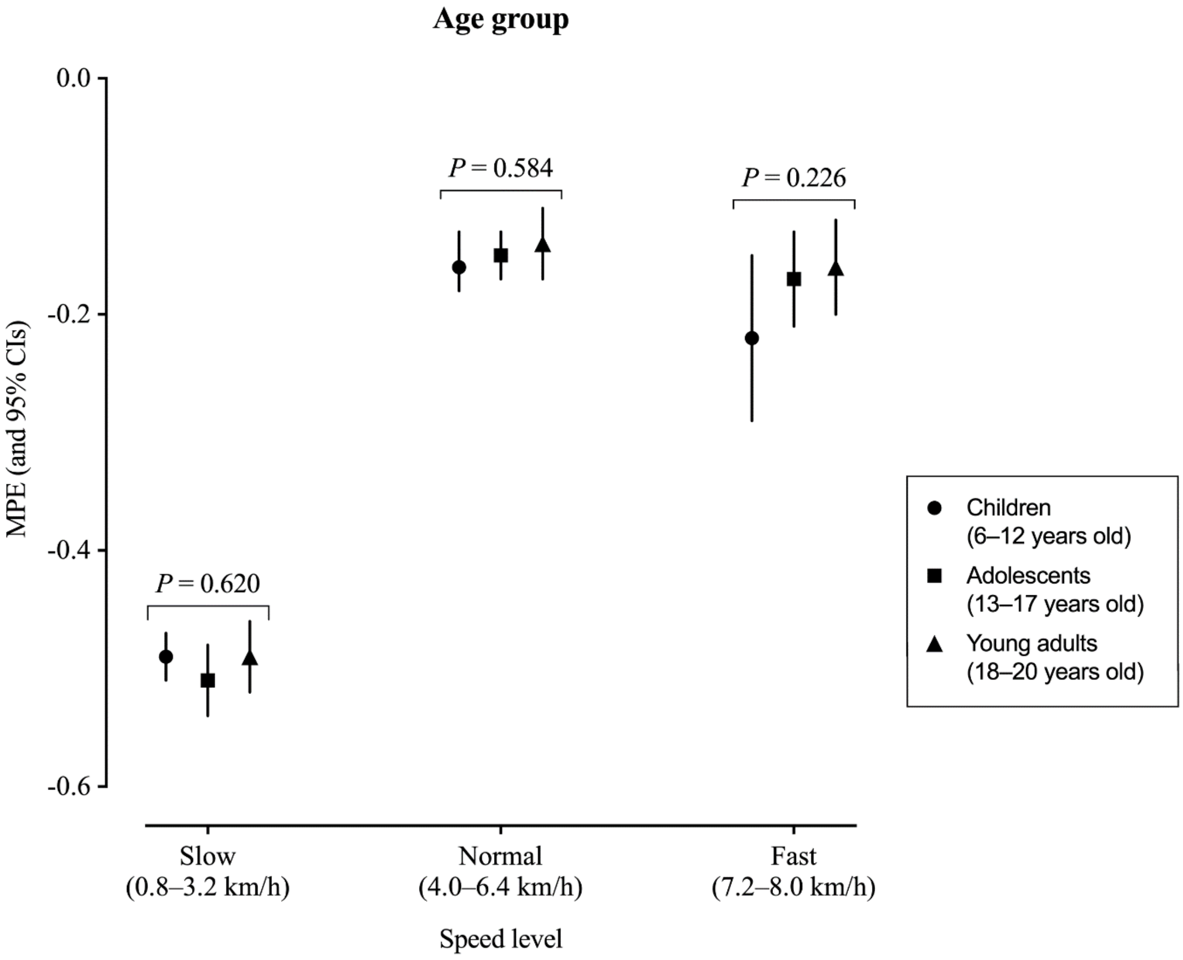

Supplementary Figure 3, Additional File 9. Effect of age on bias (mean percentage error, MPE) of wearable technologies' step counting ability. MPE and corresponding 95% confidence intervals (CIs; estimated using mixed effect models) of each age group are presented at slow, normal, and fast walking speeds. MPE values were averaged across devices respective to each age group for slow, normal, and fast walking speeds. MPE values closer to 0% indicate improved bias and negative values mean undercounting. Further, where 95% CIs do not overlap, there are significant differences between locations. Likelihood ratio test *P* value is reported for the effect of age on MPE for each specific speed level.
